# Critical dependence of morphodynamic models of fluvial and tidal systems on empirical downslope sediment transport

**DOI:** 10.1038/s41467-019-12753-x

**Published:** 2019-10-25

**Authors:** A. W. Baar, M. Boechat Albernaz, W. M. van Dijk, M. G. Kleinhans

**Affiliations:** 1 Department of Physical Geography, Faculty of Geosciences, Utrecht University, Princetonlaan 8A, 3584CB Utrecht, The Netherlands; 2 Present Address: Energy and Environment Institute, University of Hull, Hull, HU6 7RX UK

**Keywords:** Geomorphology, Hydrology, Sedimentology

## Abstract

The morphological development of fluvial and tidal systems is forecast more and more frequently by models in scientific and engineering studies for decision making regarding climate change mitigation, flood control, navigation and engineering works. However, many existing morphodynamic models predict unrealistically high channel incision, which is often dampened by increased gravity-driven sediment transport on side-slopes by up to two orders of magnitude too high. Here we show that such arbitrary calibrations dramatically bias sediment dynamics, channel patterns, and rate of morphological change. For five different models bracketing a range of scales and environments, we found that it is impossible to calibrate a model on both sediment transport magnitude and morphology. Consequently, present calibration practice may cause an order magnitude error in either morphology or morphological change. We show how model design can be optimized for different applications. We discuss the major implications for model interpretation and a critical knowledge gap.

## Abstract (web summary)

Morphological development of fluvial and tidal systems is increasingly forecasted by models, with most of them predicting unrealistically high channel incision. Here, the authors point out reasons why and suggest improvements.

## Introduction

River valleys, coastal plains, and deltas are changeable landscapes with a large part of the human population that will be at risk from climate change effects and sea level rise. Adaptation requires a system approach^1,2^ with combinations of hard engineering measures and sediment attrition^3^. Reliable forecasting of effects of combined measures requires morphodynamic models for rivers, estuaries, deltas, and coasts. Morphodynamic models are therefore widely used tools to study and forecast the development of these landscapes. However, in practice, all large-scale models depend on model choices and need some form of calibration to converge to a stable morphology, for example by the choice in roughness predictor^4,5^, adding coarser grain sizes in the channels^6^ or include a non-erodible layer that limits channel depth^7^, and increasing the transverse bed slope parameter, which determines the amount of sediment transported on channel side slopes. The latter has proven to be most effective, since the bed slope parameter linearly increases downslope sediment transport and thereby directly affects channel depth and bar dimensions and therefore has the largest effect on large-scale morphology^8,9^.

The problem is that morphodynamic models show severe and unrealistic channel incision and require artificially and seemingly arbitrarily transverse bed slope parameters up to a 100 times higher^8–10^ than physically correct^11–13^ to counteract this incision and obtain realistic bar and channel patterns. A recent comprehensive set of experiments showed that a physically realistic value for the slope parameter is in the order of one and a realistic calibration range is within a factor of two^13^. This calibration range is therefore much smaller than needed in recent model studies. The need to apply unrealistically intense bed slope effects implies a flaw in the balance between the non-linearity of sediment transport that carves out channels and downslope sediment transport that counteracts this incision. Increasing the magnitude of downslope sediment transport by more than an order of magnitude raises doubts about the physical validity and predictive power of these models. It begs the question whether these models converge to a balance between erosion and deposition for the right reasons, whether sediment transport magnitudes can be correct at the same time, and what aspects of the forecasts on timescales of a century are most unreliable.

**Fig. 1:**
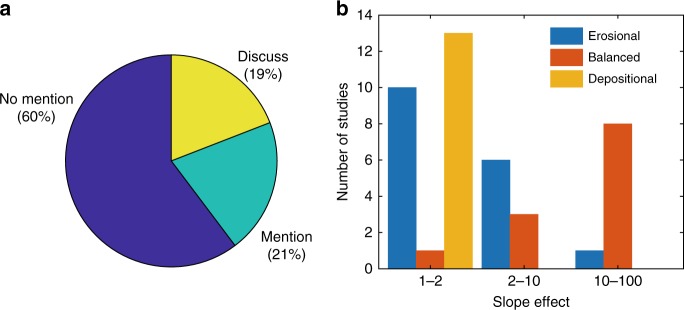
Literature inventory of slope effects in morphodynamic models. **a** Model studies that mentioned, discussed, or overlooked the severe channel incision and the artificial increase in slope effect that was necessary to counteract this (see Supplementary Information for inventory). **b** Studies that mention the magnitude of the slope effect subdivided by modeled environment and the applied slope effect value (1 = default)

The severe channel incision is not only best known for sensitive codes such as Delft3D^8,9^ but is also an issue in studies with other morphodynamic models. Studies with, for example, the Regional Ocean Modeling System or Telemac report the need of a bed slope diffusion term^14^ or a coarsening of the bed^15,16^ to prevent unrealistic bed erosion and sharp morphodynamic features. An inventory in typical geomorphology journals showed that only 13 (19%) out of 68 model studies discussed the need to increase the slope effect due to the imbalance between severe incision and downslope sediment transport, and 14 (21%) studies only mentioned the magnitude of the slope parameter in their model (Fig. 1; see Supplementary Information for an inventory).

The literature inventory suggests that sensitivity to incision depends on the environmental settings (Fig. 1b). Here environment means initial and boundary conditions, which determine sediment characteristics, flow conditions, channel pattern, and bar regime. Models of environments with a large-scale balance between erosion and deposition, such as estuaries and rivers, particularly have the tendency to overpredict channel depth and number of channels and required very high slope effects up to a factor of 100^5,17^. In contrast, models of systems with dominant erosion such as a tidal channel network, usually had slope factors <10^18–20^, and depositional systems such as river deltas all used the default value^21–23^. However, increasing the slope effect to obtain realistic channel depth and bar dimensions results in an unrealistically large downslope sediment flux, which determines the rate of bank erosion, channel formation, and migration. On the other hand, default transverse slope parameters in both erosional and depositional models commonly show unrealistically deep channels and sharp angular bends^23–26^. While these angular bends have been attributed to grid resolution, we here show that the underlying cause is in the sediment transport.

**Fig. 2:**
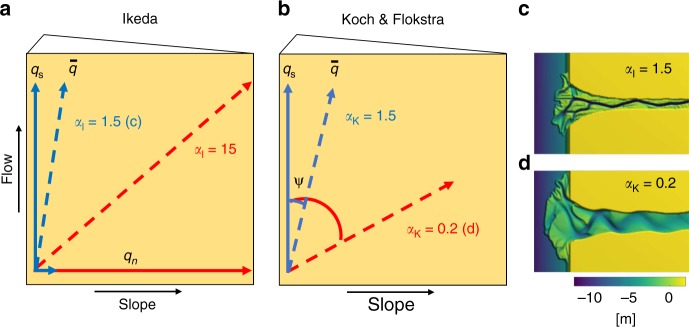
Schematic drawing of the two main slope parameterizations. The parameterizations for sediment deflection by Ikeda^27^ (${\alpha }_{{\mathrm{I}}}$) and Koch and Flokstra^28^ (${\alpha }_{{\mathrm{K}}}$) drawn on a transverse bed slope. Both methods are drawn on a top view of a bed sloping toward the right. Blue solid arrows show sediment transport in streamwise direction (${q}_{{\mathrm{s}}}$) and transverse direction (${q}_{n}$, only for Ikeda), and dashed blue arrows show the resulting transport vectors ($q$) with default values for the slope effect. Red arrows represent transport vectors when the slope effect is increased to typical values used in current model studies. **a** The method of Ikeda increases the transverse sediment vector as a function of slope and ${\alpha }_{{\mathrm{I}}}$ and thereby increases the resulting sediment transport vector. **b** The method of Koch and Flokstra rotates the streamwise transport vector over an angle ($\psi$) as a function of slope and ${\alpha }_{{\mathrm{K}}}$. ${\alpha }_{{\mathrm{K}}}$ is roughly the inverse of ${\alpha }_{{\mathrm{I}}}$. See Supplementary Note 1 for detailed calculation method and how to translate ${\alpha }_{{\mathrm{K}}}$ into ${\alpha }_{{\mathrm{I}}}$. **c**, **d** Examples of a modeled river delta for default (${\alpha }_{{\mathrm{I}}}$ = 1.5) and high (${\alpha }_{{\mathrm{K}}}$ = 0.2) slope effect (see Supplementary Fig. 6 for more examples)

The use of different sediment transport predictors, which relate the sediment transport rate to flow velocity, and parameterizations for the deflection of sediment transport on transverse slopes reflect the present uncertainty about the non-linearity of sediment transport and the negative feedbacks on run-away deepening. The frequently used sediment transport predictor of Engelund–Hansen (EH), which relates sediment transport rate to flow velocity to the power of 5, has a higher sediment transport rate than the predictor of Van Rijn (VR), which relates sediment transport rate to flow velocity to the power of 3 for high mobility and much higher powers for lower mobility. Many other relations for bed load transport have qualitatively similar behavior. The predictor of VR furthermore makes a distinction between sediment transported over the bed and in suspension and assumes that the bed slope effect only acts on the bed load part. As a result, the predictor of EH will deflect more sediment downslope than the predictor of VR and similar relations at the same flow velocity. The two most commonly used slope parameterizations, by Ikeda^27^(IK) and by Koch and Flokstra^28^(KF), calculate the downslope sediment transport vector differently (Fig. 2). For KF, the streamwise transport vector is rotated as a function of the transverse bed surface gradient, while for IK the normal transverse transport vector is enhanced before combination with the streamwise transport vector. As a result, the method of IK not only changes the direction but also increases the flux of sediment transport. How this affects morphology and the rate of change thereof remains unquantified. Most other bed slope parameterizations have similar behavior to one of the aforementioned^13^.

**Fig. 3:**
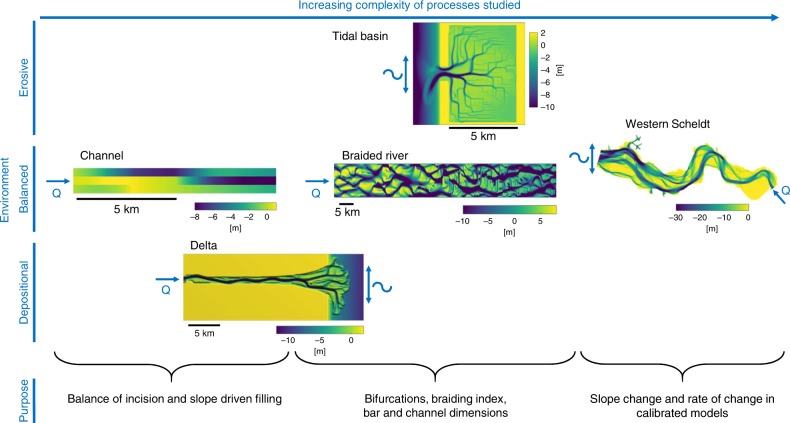
Overview of model environments and objectives in this study. The narrow channel model and the river in the delta model are used to study local sediment transport processes. The delta model, the braided river model, and the tidal basin model quantify the effects of different slope parameterizations and magnitudes in combination with different sediment transport predictors on bifurcation dynamics, braiding index, and channel dimensions. The calibrated Western Scheldt estuary model shows differences in dynamics between models with different slope parameterizations and magnitudes relevant for fairway dredging depth and intensity

Here we conduct five sets of numerical morphodynamic simulations for different scales and environments, i.e., erosional, depositional, or balanced, to quantify the effects of increased downslope sediment transport on morphology (Fig. 3). The flow velocities in all models are such that suspended sediment transport of sand plays a significant role, but we do not consider suspension of cohesive sediments. The objective is first to identify possible causes of the imbalance between incision and transverse sediment transport on the channel scale for typical combinations of sediment transport and slope parameterizations. Second, we quantify the effects of local sediment transport vectors on large-scale morphology of rivers, estuaries, and deltas. Finally, we will discuss sensitivity to environment and the large range in slope effect that is applied between different model studies and consequently give recommendations for an appropriate design of models depending on research objectives of future studies given the present limitations and uncertainties.

## Results

### Effects of slope parameterization on general morphology

**Fig. 4:**
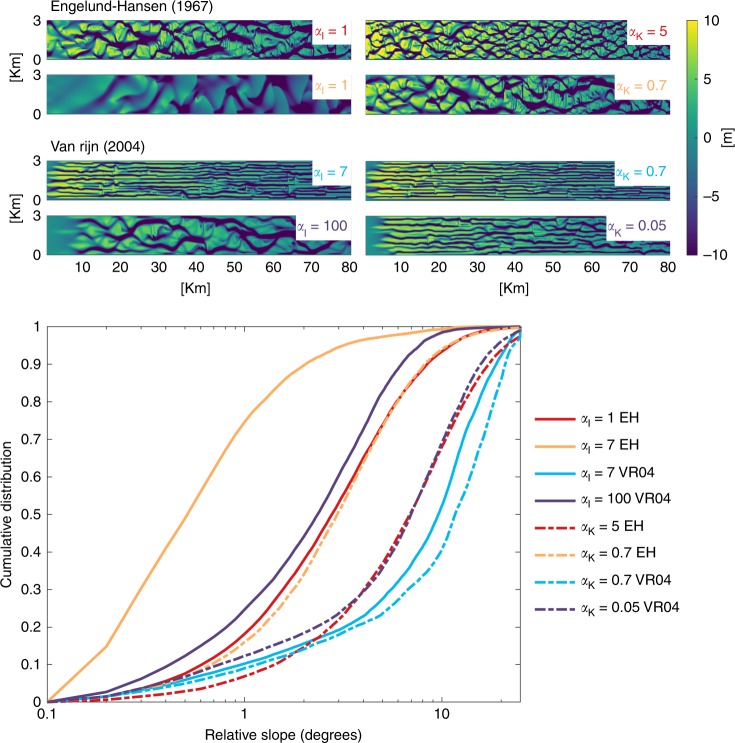
Influence of slope effect and transport predictor on morphology. Morphology of eight braided river model runs for different combinations of slope effect and sediment transport predictors. Models on the horizontal axis have equal slope effect. The ${\alpha }_{{\mathrm{I}}}$ is the input parameter of the method of Ikeda^27^, while the ${\alpha }_{{\mathrm{K}}}$ is the input parameter of the method of Koch and Flokstra^28^, both with defaults of order 1. The graph shows the cumulative distribution of the slopes of all grid cells in the same models at the same timestep. Solid lines are results with IK and dashed lines are results with KF. Colors indicate equal transverse sediment transport magnitudes and the same sediment transport predictor

All five models (Fig. 3) generally showed deep incision and steep morphology with physically correct slope effects, leading to deep and narrow channels, a higher number of channels, and shorter bars than typically observed in nature. Increasing the bed slope effect resulted in wider and shallower channels, longer bars, a smaller braiding index, and fewer bifurcations and a greater similarity to natural systems, although a very high slope effect caused overly subdued relief in models with the EH sediment transport predictor (Fig. 4; Supplementary Figs. 6–8). However, different combinations of sediment transport predictor and slope parametrization led to starkly contrasting morphologies. To quantify the difference in effect of both slope parameterizations on sediment transport processes and morphology, the parameter that determines the magnitude of the transverse slope effect was systematically increased. Henceforth, the term slope effect refers to the magnitude of this parameter, which is the ${\alpha }_{{\mathrm{I}}}$ in the method of IK and the ${\alpha }_{{\mathrm{K}}}$ in the method of KF (Supplementary Note 1). Note that the parameter ${\alpha }_{{\mathrm{K}}}$ is roughly the inverse of ${\alpha }_{{\mathrm{I}}}$. To be able to compare the differences between both options, the values for these parameters were not simply proportionately varied but determined by requiring equal sediment transport in the transverse direction as explained in Supplementary Note 1 (Supplementary Fig. 1). The Supplementary Information shows all model digital elevation models (DEM) and cumulative bed slope distributions; here we use the braided river model as an example.

Models with the KF parameterization, which merely rotates the transport vector, had steeper slopes and deeper channels than models with the IK parameterization, which calculates an additional transverse vector and therefore increases sediment transport (Fig. 4). Increasing the slope effect with the IK parameterization in models with the EH sediment transport predictor resulted in significantly lower bed slopes, while this decrease in bed slopes was significantly less than when increasing slope effect with the KF parametrization. Models with the VR sediment transport predictor had much steeper slopes and deeper channels than with EH and showed unrealistically long thin bars with the default value (Fig. 4). The most worrying conclusion is that the braided river model with the EH transport predictor and an ${\alpha }_{{\mathrm{K}}}$ of 0.7 (slope effect = 7) has similar morphology as with an ${\alpha }_{{\mathrm{I}}}$ of 1 (slope effect = 1) (Fig. 4) but has seven times larger transverse sediment fluxes on the same slope, since the slope effect is seven times larger, which also means a large change in the direction of sediment transport. The consequence is that the timescale of morphological adaptation differs considerably, which possibly has major implications for model studies that are used for management strategies. When using another sediment transport predictor, this difference is even larger, since a hundred times larger slope effect is needed in the model with the VR transport predictor to get similar bed slopes as in the model with the EH transport predictor (Fig. 4). In the wide braided river model, it was not possible to get a realistic morphology in combination with the KF slope parameterization.

### Imbalance between incision and transverse sediment transport

The unrealistic channel erosion in numerical models suggest an imbalance between channel incision and transverse sediment transport. Therefore, the overdeepening of channels can be the result of either of these two processes. To understand this imbalance, it is necessary to compare the different sediment transport predictors to the theoretical equilibrium between incision and downslope sediment transport at the channel scale. In nature, the width-to-depth ratio determines whether minor perturbations on a flat bed decay or grow into channels and bars, with the braiding index depending on the width-to-depth ratio^29^. Growing perturbations mean channel erosion. This is caused by the non-linear dependence of the sediment transport rate on flow shear stress at the bed, so that deeper channels that attract more flow have disproportionally more sediment transport capacity that is not balanced by the upstream supply of sediment. This positive feedback is strongest near the critical flow velocity for sediment motion, where the non-linearity of sediment transport is largest and therefore tends to deepen channels, albeit at a low rate. The most important negative feedback on channel formation is sediment transport deflection on the side slopes toward the center of the channel under the influence of gravity^12,30,31^, which is thus a crucial feedback in forming equilibrium channels. Wider and shallower channels tend to incise more, so that larger bed slope effects are needed to prevent deepening of channels, and this equilibrium determines the development of bars and sets the braiding index^29,32^. The transition between decay and growth of a perturbation is therefore a function between width-to-depth ratio and the transverse sediment flux and can be analytically described.

To determine the tendency to incise independently of numerics, we use an analytical model of a river channel cross-section, which is described in Supplementary Note 2. This analytical model calculates the theoretical equilibrium width-to-depth ratio of the channel. Channels with lower ratios should theoretically show decaying perturbations, while models with higher ratios should have growing perturbations (Supplementary Fig. 2). The equilibrium width-to-depth ratio depends on the non-linearity of the sediment transport predictor and the magnitude of the slope effect, since incision is more dampened when more sediment is transported toward the channel center (Supplementary Figs. 3 and 4). For the sediment transport predictor of VR, we only take the bed load part into account in the analytical model, since in Delft3D slope effects only act on the bed load. This analytical model is compared to a very simple numerical model scenario of three grid cells wide. This prevents formation of complex patterns so that channel and bar formation are fundamentally the result of the balance of two processes: channel erosion and gravity-driven sediment motion toward the channel center. Channel width was varied between 21 and 210 m.

With the default value for the slope effect (${\alpha }_{{\mathrm{I}}}$ = 1.5), the VR models corresponded reasonably well with the analytical model, since the transition from a dampened system toward a channel where the perturbation grows is around the theoretical equilibrium line (Supplementary Fig. 5). However, the numerical models with increased slope effect significantly deviated from the analytical model. These models required a disproportionately larger slope effect to dampen the initial perturbation (>30 times higher than the default factor as opposed to 4 times the default in the analytical model). On the other hand, the initial perturbation in models with the EH predictor immediately decayed (Supplementary Fig. 5) until the channel has a width-to-depth ratio around 36, which is >15 times higher than the theoretical model. These results demonstrate a stronger tendency to incise in the numerical model with VR than expected from theory and a weaker tendency to incise in numerical models with EH.

**Fig. 5:**
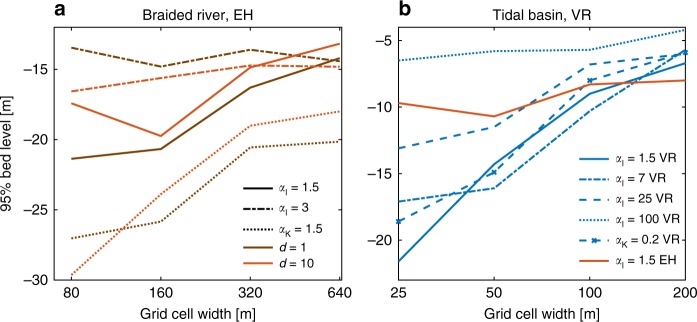
Grid size-dependent incision. Tendency to incise quantified as 95% depth against size of the grid cells for different magnitudes of slope effect, of **a** the braided river model in combination with the sediment transport predictor of Engelund–Hansen, and a horizontal eddy diffusivity $d$ of 1 or 10, and **b** the tidal basin model

However, all large-scale morphodynamic models show unrealistic channel incision independently of sediment transport predictor and need increased slope effects to balance this^8^ (Fig. 4; Supplementary Figs. 6–8), which suggest that the imbalance at the channel scale does not only depend on the sediment transport predictor and the resulting amount of transverse sediment transport but also on the rate of incision. To study whether the overdeepening of channels is a numerical issue, grid size is systematically varied for the tidal basin model and braided river model. Results show that equilibrium channel depth increases with decreasing grid size in models with a low transverse slope effect (Fig. 5). With increasing slope effect, grid size-dependent incision decreases and with a sufficiently large slope effect there is no trend with grid size. However, the transition between grid size-dependent incision and no grid size dependency differs for each sediment transport predictor and slope parametrization. The braided river model with the EH transport predictor shows this transition around a slope effect of ${\alpha }_{{\mathrm{I}}}$ = 3 (Fig. 5a), while the tidal basin model with VR needs a slope effect of ${\alpha }_{{\mathrm{I}}}$ = 100 (Fig. 5b). Furthermore, models with the KF slope parametrization again show a larger incision than models with the IK parametrization and the same slope effect. In the braided river models, also the horizontal eddy diffusivity was changed from 10 to 1, and this resulted in slightly different distributions of channel depth but did not have the same amount of influence as increasing grid size or changing slope effect (Fig. 5a).

### Effect of grid size-dependent incision on channel dynamics

**Fig. 6:**
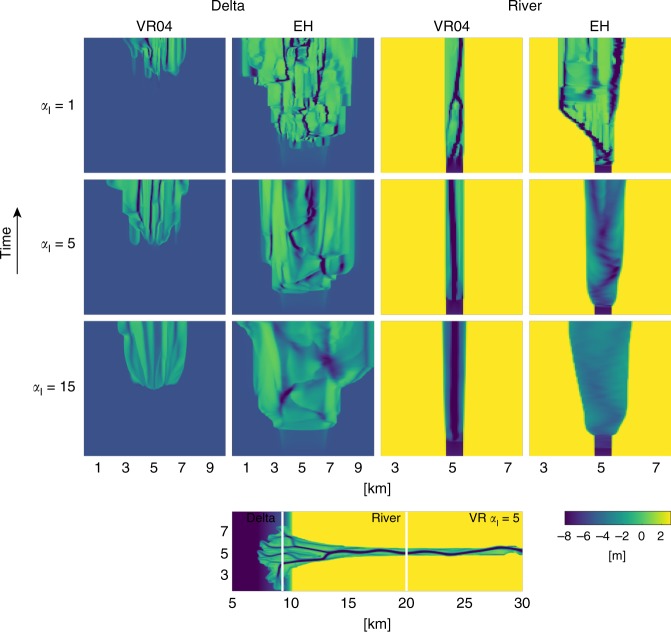
Effect of grid size-dependent incision on channel dynamics. Development of the bathymetry of a cross-section at the river and at the delta over time in the delta model, for different combinations of sediment transport predictor and magnitude of the slope parameter with the method of Ikeda^27^

Large-scale morphology critically depends on the balance between incision and downslope sediment transport at the channel scale, which is illustrated in Fig. 6. The delta model (Fig. 3) initially exists of only a straight channel, before it starts transporting sediment and depositing it in the sea basin (Supplementary Fig. 10). However, over time, the river in the models with the VR transport predictor stays within that initial channel without moving sideways, and only at a high slope parameter it starts to erode the initial banks. In contrast, models with the EH transport predictor are immediately much more dynamic. This illustrates the effect of the difference in slope effect needed to balance incision at the channel scale between both transport predictors. On the other hand, the delta is a depositional environment and depends on sedimentation instead of the non-linear incision and therefore initially does not have to erode banks. As a result, depositional models with the EH transport predictor show a subdued morphology due to the large sediment transport rates, which is enhanced with increasing slope effects. However, the channels on the delta show similar dynamic behavior as in the river part of the model, since they incised in the deposited material. Once channels start to form in the models with the VR transport predictor, their location seems to be fixed owing to the transverse sediment transport rate that is too low, while channels on the delta in models with the EH transport predictor show lateral movement and regular avulsions. Channels in the erosive tidal basin model showed the same behavior: once a channel was formed in models with the VR sediment transport predictor it was fixed to that location, while channels in models with EH were still able to migrate (Supplementary Fig. 9). This difference in channel dynamics shows that the model has to overcome extreme incision at the channel scale by increasing slope effects to model a dynamic system. Only when grid size-dependent incision is balanced by downslope sediment transport, the channel can migrate sideways.

### Slope effect-dependent bar and channel properties

The environment that is modeled, i.e., depositional, erosive, or balanced, controls the growth or decay of perturbations at the channel scale and therefore influences how likely models are to incise and how sensitive they are to changes in bed slope effect. We now quantify effects on bars and the degree of braiding, which are critical elements of fluvio-deltaic patterns. Here the delta in the delta model is a perfect depositional environment. The braided river model represents a Brahmaputra-sized braided sand-bed river with a 3.2-km wide and 80-km long braidplain where erosion and deposition are on average in balance and is exactly the same as the model of Schuurman and others^9^. The tidal basin model consists of a channel network that is incised by the tidal motion, and therefore this model represents an erosional environment (Fig. 3).

Downslope sediment transport counteracts incision but also balances effects of helical flows in curved channel sections. In nature, secondary currents alter the direction of the bed shear stress toward the inner bend, which leads to a balance between the upslope directed drag force by the secondary flow and the downslope sediment transport under the influence of gravity^29,33^. By balancing secondary flows, downslope sediment transport controls the adaptation of the bar morphology to spatial gradients in flow conditions and along meanders^29,32,34^. Therefore, by both counteracting incision and balancing secondary flow, the magnitude of downslope sediment transport determines the developed active channel width, orientation of channels, and the length and migration rates of fluvial and tidal bars^9,19,35,36^ and controls the division of bed load over bifurcates^34^. On the larger scale, the amount of downslope sediment transport therefore has a major influence on channel and bar patterns by determining braiding index^29,32,37^ and the stability of river bifurcations and related tendency of channels on fans and deltas to avulse^4,38–40^.

**Fig. 7:**
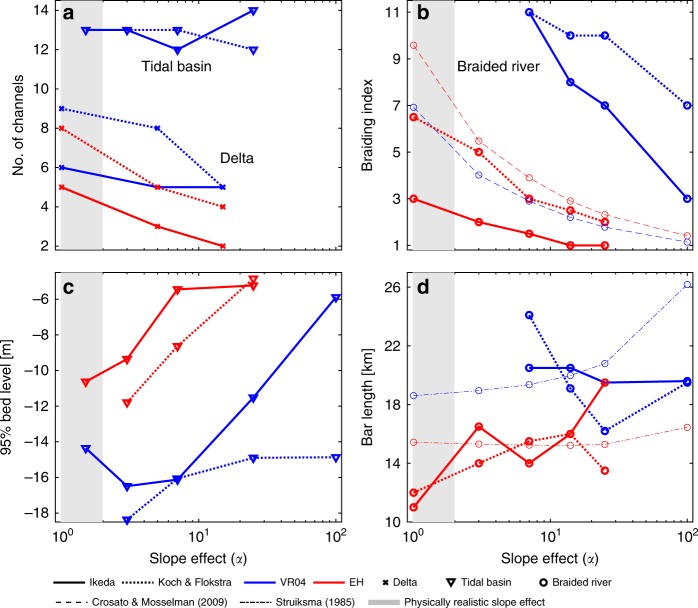
Relation between slope effect and morphodynamic element dimensions. **a** Number of channels at the delta front in the delta models, and the number of channels in the tidal basin models, against increasing slope effect. **b** Braiding index with increasing slope effect in the braided river model, with the semi-analytical predictor for braiding index from Crosato and Mosselman^32^ in corresponding colors for comparison. **c** The 95% depth of all tidal channels in the tidal basin models against slope effect. **d** Mean bar length in the braiding river model when increasing the slope effect, including the predictor of Struiksma and others^29^ for wave lengths of bars. Braiding index and bar length are computed according to the methods described in Schuurman et al.^9^. The method of determining the number of channels in the tidal basin models is explained in the “Methods” section. Slope effect is given as the ${\alpha }_{{\mathrm{I}}}$ for IK and transformed for KF

Braided river models with the KF slope parameterization had a larger braiding index and shorter bars than the models with the IK slope parameterization with the lowest slope effect, and the braiding index decreased with increasing the slope effect for both sediment transport predictors (Fig. 7b). Models with the EH sediment transport predictor showed braiding indices that were lower than predicted with the braiding index predictor of Crosato and Mosselman^32^, especially at a lower slope effect. However, models with the KF slope parameterization had braiding indices that were only slightly lower with a higher slope effect than the braiding index predictor and generally showed the same trend in decreasing braiding index with increasing slope effect. Models with the VR sediment transport predictor theoretically should have lower braiding indices due to the lower non-linearity of sediment transport, but in these models many deep and narrow channels developed separated by long bars (Fig. 4). Only with downslope sediment transport that was almost a hundred times larger than with the default value, realistically shaped bar patterns developed, but the braiding index was still too high.

In general, bar length in the braided river model increased with increasing slope effect in models with the EH sediment transport predictor, but for the models with the IK slope parameterization bar length showed a local decrease with an intermediate slope effect (Fig. 7d). For strong slope effects, a subdued morphology is visible with short and wide bars. Bar length also decreased slightly in the model with the KF slope method and with the largest amount of downslope sediment transport. Predicted bar length by Struiksma and others^29^ is in the same range as the models but show a much more stable bar length with an increase in slope effect. As a result, bars in the braided river model are theoretically longer when the slope effect is weak and shorter when the slope effect is strong. Models with the VR predictor showed a decreasing bar length with increasing slope effect, since here cross bar channels started to dissect the unnaturally long bars separating the deep channels or started to show realistically shaped bar patterns in the case of the model with the IK slope parameterization. The bar length predictor predicts increasing bar lengths with increasing slope effect, which is therefore not comparable with bar lengths in the braided river models with the VR predictor.

The number of avulsions in the delta models is larger for runs with the KF bed slope predictor compared to runs with the IK parameterization, even though sediment transport rates were equal for models with an equal downslope sediment transport. Models with VR had a larger number of avulsions compared to models with the EH predictor with equal slope effect (Fig. 7a). In contrast, the tidal basin model shows that the number of channels is not significantly affected by increasing downslope sediment transport in an erosive environment (Fig. 7a). Furthermore, the amount of incised channels was also similar between models with different slope parameterizations. The magnitude of the bed slope effect did have an influence on channel dimensions, since in general channels became shallower with increasing downslope sediment transport (Fig. 7c).

## Discussion

The extreme incision common in morphodynamic models is the result of an imbalance at the channel scale between the non-linearity of sediment transport that carves out channels and transverse sediment transport that counteracts incision. The cause of this imbalance is twofold. First, the amount of channel incision is highly depended on grid size, suggesting strong numerical effects. When a channel incises, the channel will attract more flow and will experience a positive feedback. The flow seems to prefer flowing through as few grid cells as possible, and when grid cell width is smaller this means that there is more discharge flowing through a smaller area, which therefore results in more incision. The discharge flowing through a much smaller area than a natural channel width results in an unrealistically deep channel at equilibrium. Lateral channel migration requires erosion and movement of all sediment in the high banks, so that deep channels are effectively unable to migrate sideways. The transition from grid size-depended incision to a more dynamic system is determined by the transverse sediment transport rate and can therefore be reached by increasing the transverse slope parameter (Fig. 5). Some studies suggest the severe incision is caused by the use of uniform sediment instead of a sediment mixture, which would lead to coarser sediment to be deposited in the deeper parts and therefore a reduce in flow velocities^6,41^. However, for realistic grain size mixtures active sediment sorting will not lead to different transverse slopes^42^. Our results show that the extreme grid size-dependent incision with uniform sediment is not natural, and therefore, adding coarser sediment fractions to the model does not solve the problem of severe incision but can mask it by resulting in a non-erodible bed layer that prevents erosion.

Second, the magnitude of slope parameter that is needed to overcome grid size-dependent incision is determined by the bed load transport rate that is initially available for deflection downslope. This transport rate is calculated by the sediment transport predictor, which determines both the sediment transport rate and the ratio of bed load versus suspended load. Simple transport predictors such as EH overdampen perturbations due to the high total sediment transport rate and because slope effects act on all sediment transport. On the other hand, VR initially predicts the correct balance between incision and downslope transport in accordance with the analytical model (Supplementary Fig. 5). However, once incision commences, it needs much higher slope effects to counteract incision than in theory. This can be explained by the distinction of suspended and bed load transport, since VR and similar suspended load predictors assume that bed slope effects only act on bed load. Additional bed slope effects on suspended sediment and the influence of the vertical distribution in the water column are not accounted for^31,43,44^. Consequently, the tendency to incise depends on grain size and sediment mobility, since this determines the amount of sediment that is transported in suspension^6,9,45^. More suspension means that there is less bed load available for deflection downslope and therefore leads to a higher slope parameter to counteract incision. However, there are some model studies with only suspended sediment or very high suspended sediment concentrations that do get realistic channel morphology. This can be explained by large numerical diffusion^46^, dampening of the turbulence near the bed due to large suspended sediment concentrations^41^, or by modeling a small and constrained domain with well-defined boundary conditions^47,48^. Therefore, it is advised to further study the role of slope effects on, and diffusion of, suspended sediment transport by modeling and experiments^49^.

To model a dynamic system, the model has to overcome extreme incision at the channel scale by increasing the transverse bed slope effect. The magnitude of the transverse slope parameter that is needed depends on whether a model needs to be laterally dynamic or not and thus whether it has to overcome the grid size-dependent incision. Therefore, the difference in slope factor that is used in dynamic, erosional, or depositional systems in previous model studies (Fig. 1) is also explained by the research objective, next to flow conditions and the choice in sediment transport predictor. Environments with a large-scale balance between erosion and deposition, such as estuaries and rivers, particularly have the tendency to overpredict channel depth and braiding index and require very high slope effects to overcome the severe incision and show realistic morphology. The initial response determines whether a system tends to incise or goes toward an equilibrium channel with a constant width-to-depth ratio by eroding the banks. Once a channel incises, it attracts more flow and will deepen further through the aforementioned positive feedback, and therefore especially models with weak slope effects had a more extreme deviation in channel depth, braiding index, and bar length compared to theory (Fig. 7). In case of erosional models, in some studies bank erosion was calibrated and therefore slope effects were increased, which will set channel dimensions but not necessarily the number of channels (Fig. 7). However, the majority of the models presumably only focused on the network characteristics and therefore saw no need to increase slope effect. As a result, many studies show unnaturally sharp angular bends in plan view. These angular bends that are observed in many models especially with the transport predictor of VR are also explained by grid size-dependent incision and the resulting lack of channel migration. The channels follow the grid configuration, which is rectangular in this study, and therefore it is expected that models with an irregular shaped grid will show other bend shapes, but this does not mean that the problem of grid size-dependent incision and lack of channel migration is solved in this case. In contrast, depositional models like the delta model will show more natural looking bars with default slope parameters (Supplementary Fig. 6), since deposition does not depend on the non-linearity of sediment transport that carves out channels. In this case, increasing the slope effect would quickly lead to a diffuse morphology. However, channels that form on the deposits will incise during the model run and often show the same rectangular bends as in the erosional models, as observed in previous model studies^23,24,26^.

The slope parametrization determines local direction of sediment transport and thereby the magnitude of downslope sediment transport. The IK slope parameterization increases the total sediment transport by calculating an additional transverse transport vector, while the KF slope parameterization only causes a larger rotation of the transport vector. This difference between slope parameterizations in direction and magnitude of the transport vector significantly influences the development of morphology across scales (Figs. 4 and 7). The larger magnitude of the total transport vector in models with the IK slope parameterization results in wider and shallower channels. The larger rotation of the transport vector in models with the KF slope parameterization results in a different distribution of sediment over bifurcates and a shorter adaptation length to changes in flow conditions, influencing bifurcation dynamics and bar dimensions. Since both slope parameterizations distribute sediment differently, this also modifies channel curvature and therefore the orientation of channels at bends and bifurcations. This orientation affects locations of bank erosion, migration rate, and chute cutoff processes^36^. On the larger scale, this alters the timescale of morphological adaptation and the frequency of avulsion^34^ and therefore has a large influence on the development of channel patterns.

**Fig. 8:**
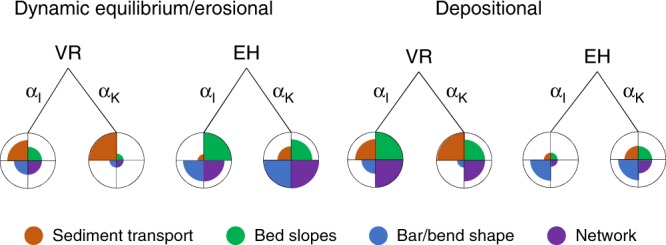
Model design recommendations. Relative performance for each combination of transport predictor and slope method in models of either erosive or balanced environments where bank erosion is necessary or depositional environments. Relative performance is divided into four categories, such that the choice of predictors can be made depending on the research objective. Network characteristics include braiding index and the number of channels in, e.g., a tidal basin or a delta

The local balance between channel incision and downslope sediment transport has a large effect on sediment transport rates, bar and channel dynamics, and consequently large-scale morphology. Therefore, modeled morphology heavily depends on the combination of sediment transport predictor and slope parametrization. Pending further investigations into sediment transport parameterizations and numerical effects, the choice of sediment transport predictor and slope parametrization in future studies should depend on the environment that is modeled and the research objective, instead of arbitrary choices. Our recommendations based on the results of this study are summarized in Fig. 8 and are not a solution but a way to limit unintended artifacts until the real problems are solved. These recommendations qualitatively hold for any sediment transport predictor that either is a bed load or total load predictor like EH or makes a distinction between bed load and suspended load like VR. Quantitatively, however, the predicted sediment transport rate and dimensions of morphodynamic features will depend on the non-linearity of the transport predictor and other predictor-specific parameters. Increasing the transverse bed slope effect leads to physically unrealistic sediment transport vectors^13^ but to more realistic bed slopes, channel depths, channel dynamics, and bar patterns (Fig. 7). Practically, this means that it is impossible to have both realistic sediment transport vectors and morphology in the same model study, and the choice of sediment transport predictor and slope parametrization depends on whether the objective is related to sediment transport processes or to channel and bar patterns (Fig. 8).

The predictor of EH will lead to more realistically shaped bars and channel networks in systems where lateral dynamics are essential, since it needs much lower slope effects to counteract the severe incision than VR. However, sediment transport rates are too high, which overdampens perturbations. Therefore, if the objective is to have realistic sediment transport vectors and morphological adaptation, the predictor of VR works better. Furthermore, since this predictor makes a distinction between bed load and suspended load, it is the only option in models where suspended sediment is essential, for example, in models of tidal environments with waves, varying flow directions, or cohesive sediments. In depositional environments where lateral dynamics are initially less important, the use of the predictor of EH is difficult due to the diffusive nature of this predictor. This predictor should only be used if the channels that eventually form have to be dynamic. The choice of slope parametrization depends on the sediment transport predictor. In models with EH, the best option is generally the KF parameterization, since this resulted in the most realistically shaped bars and braiding index^29,32^ (Fig. 7). The IK slope parameterization will lead to even more subdued morphology (Fig. 4, Supplementary Fig. 6), since the increase in total sediment transport with increasing slope parameter will further overdampen perturbations. In models with VR, on the other hand, it is advisable to use the IK slope parameterization, since this is often the only way to counteract the higher incision rate. However, it should be noted that any model with slope parameters higher than ${\alpha }_{{\mathrm{I}}}$ = 5 in case of the IK slope parameterization or lower than ${\alpha }_{{\mathrm{K}}}$ = 0.5 for the KF slope parameterization for certain do not produce realistic sediment transport rates and direction, according to the range in experimental results.

In case models are designed to represent existing morphology for, e.g., decision making or case studies, the effect of slope parametrization might seem less obvious due to often smaller or constrained model domains and shorter run time. Furthermore, the model runs start closer to the desired equilibrium with the existing flow conditions than when starting from incipient formation of channels as in the models discussed so far. However, starting with close-to-equilibrium morphology does not affect the final morphology and the model will incise when this is not balanced by an increased slope effect. This means that morphological models cannot produce more than one equilibrium morphology based on the initial conditions. This is illustrated by the Western Scheldt model, which started with measured bathymetry (Supplementary Note 3). After 10 years, default values of the slope effect lead to slopes that are too steep even though the model started from the measured morphology and calibrated hydrodynamics (Supplementary Fig. 8). When the model is run for longer, the slopes start to steepen further and experience the positive feedback that leads to unrealistic incision. On the other hand, with a sufficiently high slope effect and starting with a plane bed or a measured bathymetry, the same reasonable morphologies were obtained after centuries by van der Wegen and Roelvink^8^. Furthermore, local direction and magnitude of total sediment transport in calibrated models still critically depend on the choice of sediment transport predictor in combination with the slope parameterization. When the model is calibrated on bed slopes or the shape of morphological features, different slope parametrizations will lead to a different magnitude and direction of the transport vector on the same slope (Supplementary Fig. 11) and therefore leads to different local channel dynamics, such as bank erosion rates and location of erosion and deposition. For calibrated models, it means that, when a model is calibrated on morphology but used to make an estimate of timescales of erosion or sediment migration, these estimates will depend on the choice of slope parametrization. This is, for example, the case in models of existing estuaries that are used for dredging and dumping strategies, like the model of the Western Scheldt in Supplementary Note 3. When the objective is to determine timescales of erosion or sediment migration, it is better to calibrate the model on, for example, migration rates of channels instead of bed levels. On the other hand, when models are calibrated to sediment transport timescales, morphology and bed slopes will differ between different methods. These are, for example, models that focus on the migration rate of dumped sediment, the sediment distribution at bifurcations, or the rate of bank erosion. Therefore, when models are calibrated by increasing downslope sediment transport, either sediment transport magnitude or bed slopes match to measured data, while both is not possible.

Finally, idealized model scenarios are frequently used to study fundamental morphological behaviors under controlled conditions in wide-ranging environments^9,19,23,26,50^, but the above demonstrated that conclusions from model-only studies are sensitive to a priori model choices. This shows a need for the use of converging evidence from complementary physical experiments and field data analyses.

## Methods

### General model description

The morphodynamic modeling package DELFT3D FLOW2D3D version 6.02.13.7658 was used in all models in this study. For all models, the depth-averaged version with parameterization of secondary flow was used. The sediment mobility in all models is such that suspended sediment transport of sand is important, but cohesive sediments are not considered, since this requires many more processes such as flocculation, hindered settling in near-bed fluff layers, cohesion, and salinity effects. Below, we describe the set-up of each model in detail. In Table 1, the physical and numerical parameters of interest are summarized for all five models.

**Table 1:** Overview of the default physical and numerical parameters of interest for all five Delft3D models used in this study

Model	Channel	Braided river	River delta	Tidal basin	Western Scheldt estuary
Environment	Balanced	Balanced	Depositional	Erosive	Balanced
Boundaries	River	River	River, tides	Tides	River, tides
Grain size [mm]	0.5	0.2	0.25	0.125	0.2
Roughness coefficient	*C* = 40	${k}_{{\mathrm{s}}}$ = 0.15	*C* = 50	*C* = 50	*n* = 0.022–0.028
Time step [min]	0.1	0.1	0.5	0.5	0.25
Morphodynamic run time [year]	0.33	2	1000	200	10
MorFac	1	25	200	200	20
Grid size *L* × *W* [m]	7 × 7–66.67 × 66.67	200 × 80	100 × 50	50 × 50	250 × 120–120 × 50
Horizontal eddy diffusivity [m^2^/s]	10	10	10	10	10

### Channel model

We set up a simple river channel in Delft3D for comparison to the analytical model to study the tendency to incise due to imbalance between incision and downslope sediment transport (Supplementary Note 2). This river channel has 3 grid cells across the channel, and two additional outer cells with a bed level that is 7 m higher than the inner three cells to avoid boundary effects. This means that these outer two cells are above the water level and do not interact with the channel. As a result, the active channel has the same cross-section as the analytical model (Supplementary Note 2). The discharge is equally partitioned over the three grid cells as three upstream boundary conditions.

The default model run has a channel with a length of 10 km, a slope of 0.5 m/km, a Chezy coefficient of 40 $\sqrt{m}/s$, a ratio between discharge $Q$, and channel width $W$ of $Q/W$ = 12.5 m^2^/s, and a grain size of 0.5 mm, which is all equal to the default analytical model. As a result, the average water depth is 5.8 m. The IK method is used for slope effect, with an ${\alpha }_{{\mathrm{I}}}$ of 1.5. To be able to compare the model behavior to the analytical model results, we varied the channel width between 21 and 210 m, the bed level difference of the middle grid cell and the surrounding cells between 0.01 and 3 m, and the ${\alpha }_{{\mathrm{I}}}$ between 1.5 and 50. Furthermore, we either used the VR sediment transport predictor, which relates the transport rate to the flow velocity to the power of 3 (*k* = 3) at higher mobility, or the EH predictor, where the transport rate is related to the flow velocity to the power of 5 (*k* = 5). To test whether the model results depend on the varied parameters or on the implementation of the specific transport predictor, we run the same models with the general transport predictor (Supplementary Note 1) in combination with both the IK and KF slope parameterization, with and without the critical shear stress, and varied the non-linearity between 3 and 10 (Supplementary Note 2). The models were run for 2 months, after which either the perturbation caused larger bed level differences and one grid cell-wide bars to form or the perturbation decayed and the three grid cells showed the same bed level.

### River delta model

The river delta model was inspired by the Old Rhine river mouth at Leiden, The Netherlands from the mid-late Holocene and is similar to that of Geleynse and others^51^. It consists of a 20-km-long river that flows into the coastal domain delimited as a 10 km-by-10 km sloping bed, where the sediment is deposited and a delta is formed. The river can freely migrate and forms its own topographic forcing by incising and forming meander bends. Initially, the river is a 7-m deep channel with a width of 0.5 km for the first 15 km from the upstream boundary, after which it exponentially expands over the last 5 km towards a width of 3 km at the river mouth. The sea has a depth around 4 m at the river mouth, increasing toward the end of the model domain. The upstream boundary consists of a constant discharge of 1750 m^3^/s and at the downstream water level boundary a M2 tide is prescribed with an amplitude of 0.7 m. The model is run for 5 years at the hydrological timescale with a morphological scale factor of 200, resulting in a morphological run time of 1000 years.

### Tidal basin model

**Fig. 9:**
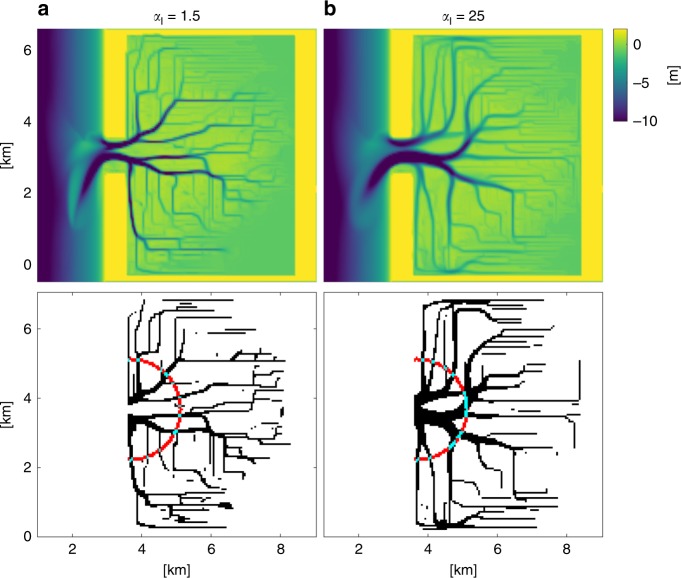
Bathymetries of the tidal inlet model. Bathymetries are shown for models with the IK parameterization in combination with an ${\alpha }_{{\mathrm{I}}}$ of **a** 1.5 (default) and **b** 25 and their corresponding binary image, where channels are black and the surrounding area is white

The tidal basin model consists of a coastal domain of 7-by-3 km and a tidal basin of 7-by-5 km, connected by a 1-km-wide inlet. The water depth at the basin is initially 1 m, and the coastal domain slopes up to 15 m depth. A 0.75-m amplitude M2 tide is prescribed at the north and south coastal boundary with a phase difference in order to create an alongshore tidal current. The initially flat tidal basin evolves with incisions due to the tidal-induced currents, promoting a rich channel network. The model is run for 12 months at the hydrological timescale with a morphological scale factor of 200, resulting in a morphological run time of 200 years. Figure 9 shows two bathymetries of model runs with the default slope parameter in the IK method (${\alpha }_{{\mathrm{I}}}$ = 1.5) and with an ${\alpha }_{{\mathrm{I}}}$ of 25. A characteristic number of channels is determined at a fixed distance from the inlet of 2.5 km.

### Braided river

The braided river model was inspired by the Brahmaputra river and is the same model that is used and described in detail in the study of Schuurman and others^9^. The model consists of a 3.2-km-wide and 80-km-long braidplain, with a slope of 0.093 mm/m. The total discharge was 40,000 m^3^/s, partitioned over 20 cells at the upstream boundary, with an initial water depth of 5 m. The initial bed and the discharge were slightly perturbated to stimulate bar development. The bed level of the upstream grid cells differed by 1 cm, and the partitioning of the discharge between the upstream grid cells varied sinusoidally through time over the cross-section, with an amplitude of 200 m^3^/s and a period of 2.28 days. In this study, the model was run for 2 years at the morphodynamic timescale.

### Western Scheldt estuary

The Western Scheldt estuary model is based on the NeVla-Delft3D schematization of the Scheldt estuary, which includes the upstream Flemish branches of the estuary, the Western Scheldt, and part of the North Sea. The NeVla-Delft3D model is a schematization from the fluid-flow behavior of the Simona simulation used by Rijkswaterstaat (the Netherlands) combined with the Delft3D component for sediment transport and morphodynamics. The NeVla model is a state-of-the-art numerical model that has been optimized for hydrodynamics^52,53^ and morphology^54,55^ and is applied by the Dutch and Belgian government.

Here we used a nested model of the NeVla-Delft3D schematization focusing on the Western Scheldt partly for reducing the computational time, which is also used by Van Dijk and others^56^. The model boundaries include the Western Scheldt from the mouth at Vlissingen to the Belgian border, in which the seaward boundary includes a water level fluctuation due to tides and the landward boundary a current. For simplification, the boundaries consist of a repeating spring–neap tidal cycles. Sediment fraction was uniform with a median grain size of 200 μm. The roughness field in the model is defined in Manning $$n$$ and is variable over the model domain, which was 0.022 s$$\cdot$$$${\mathrm{m}}^{-1/3}$$ for the eastern part, and 0.027 s$$\cdot$$$${\mathrm{m}}^{-1/3}$$ for the western part^52,53,56–58^. The bed consisted of erodible and non-erodible layers^59,60^, and therefore sediment thickness varies within the Western Scheldt model, which reduces the morphological changes but not the transverse bed slopes. To reduce computational time, the wind direction and magnitude as well as salinity were excluded because they have no effect on the transverse bed slope. We applied a morphological factor of 20 to reduce computational time and evaluated the model runs after 10 years of morphological changes.

We assessed the effect of sediment transport predictor, slope parametrization, and its calibration parameter ${\alpha }_{{\mathrm{I}}}$ or ${\alpha }_{{\mathrm{K}}}$ on the sediment transport and morphodynamics within the Western Scheldt model. The results are presented in Supplementary Information.

## Data Availability

The Delft3D models of each environment and the analytical model are available for download at 10.4121/uuid:f91de286-542b-4f9f-b6ec-f9ae7f2b8961. The analytical model is described in Supplementary Note 2. Data of the literature inventory is presented in Supplementary Table 1. Other data are available on request from the corresponding author (A.W.B.).

## Supporting Information

Supplementary Information
Peer Review File

## Supplementary information

**Supplementary information** is availbale for this paper at 10.1038/s41467-019-12753-x.

## Code availability

The analytical model is available for download at [10.4121/uuid:f91de286-542b-4f9f-b6ec-f9ae7f2b8961].
