# Supplementary material for: Critical dependence of morphodynamic models of fluvial and tidal systems on empirical downslope sediment transport
Source: Nat Commun. 2019 Oct 25;10:4903. doi: 10.1038/s41467-019-12753-x (PMC6961408; doi:10.1038/s41467-019-12753-x)
Supplement: Supplementary file 1 — Supplementary Information [file 41467_2019_12753_MOESM1_ESM.pdf]

Supplementary Information for

# Critical dependence of morphodynamic models of fluvial and tidal systems on empirical downslope sediment transport

Baar et al.

**Supplementary Figures**

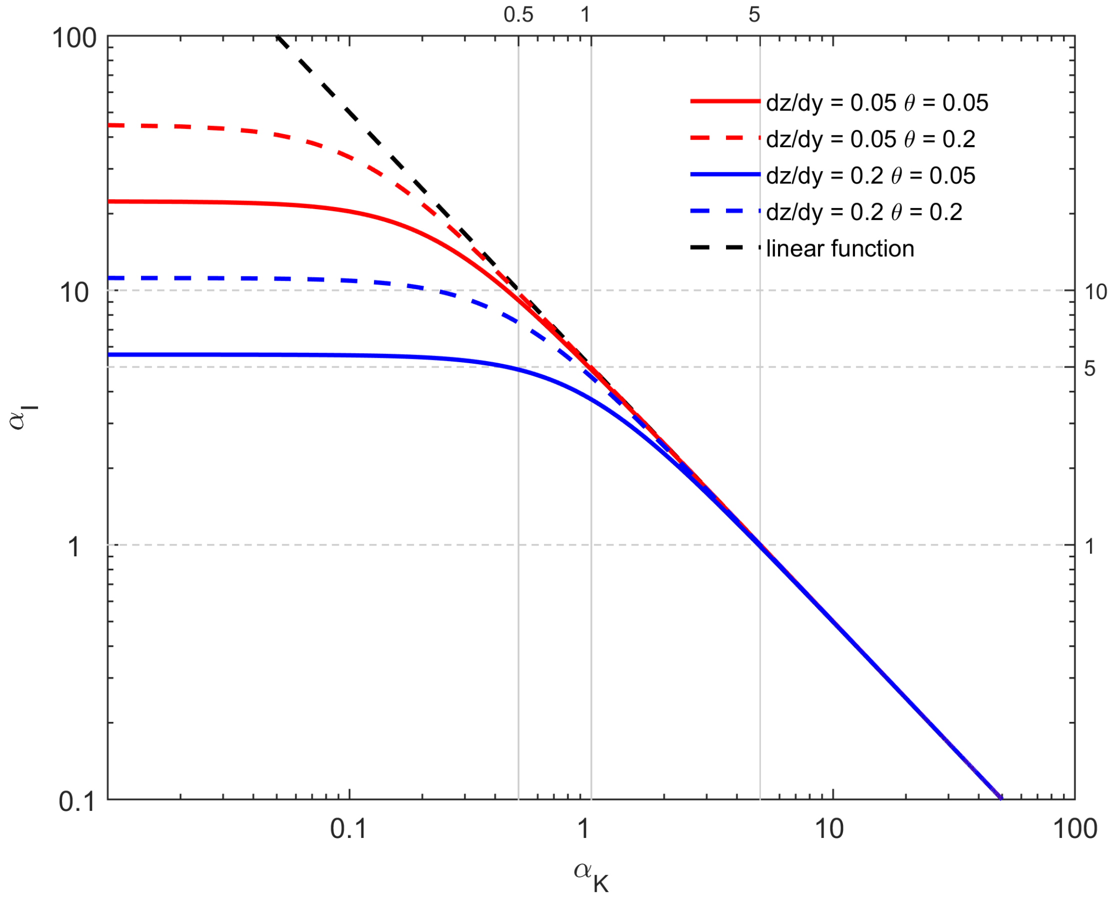


**Supplementary Figure 1:** Relation between $\alpha_I$ and $\alpha_K$, the input parameters of the two main options to calculate sediment transport on transverse bed slopes in the morphodynamic model Delft3D, when assuming equal downslope sediment transport (Eq 7). Colored lines indicate combinations of transverse slope and sediment mobility, with a critical sediment mobility of 0.04. Gray lines indicate values for the $\alpha_I$ and $\alpha_K$ used in the delta model (Supplementary Figure 6). Adapted from Baar and others[1]

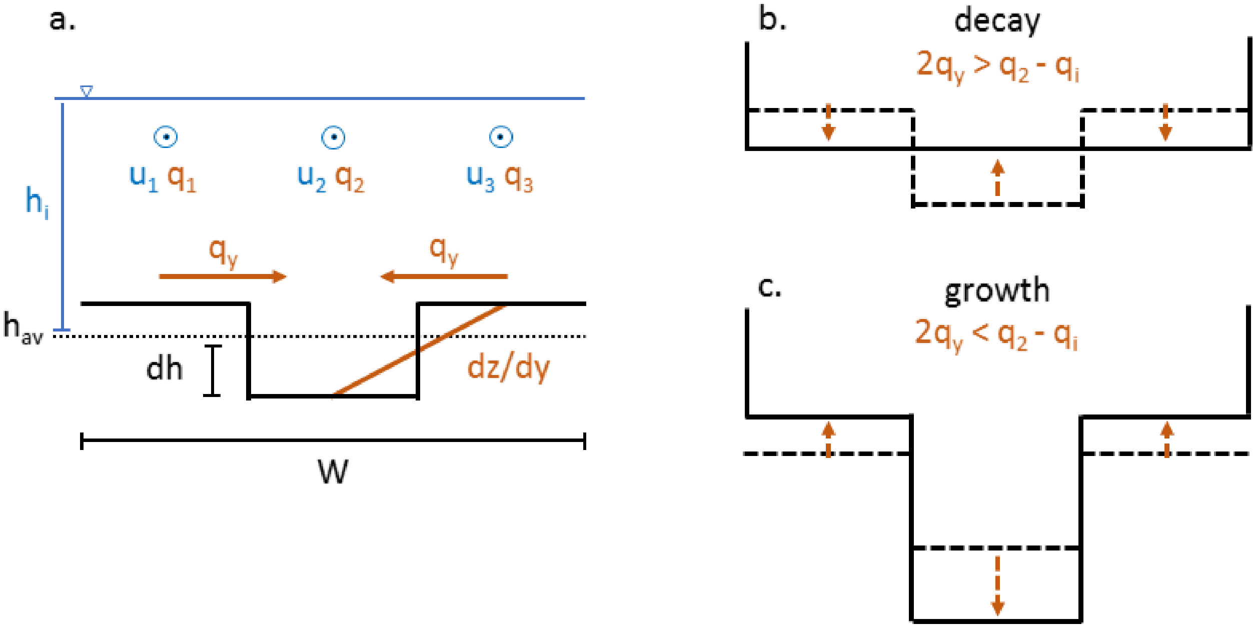


**Supplementary Figure 2:** Concept of the analytical model. The cross-section is three grid cells wide with a bed level difference between the middle grid cell and the surrounding cells as an initial perturbation. The numerical channel is also based on this concept. a) Definition of the flow velocity and transport vectors, channel width ($W$), initial channel depth ($h_i$), bed level difference ($dh$), and transverse slope ($dz/dy$). b) The perturbation decays when transverse sediment transport is larger than the difference between incoming and outgoing sediment transport. The middle grid cell will accrete, while the surrounding cells will erode till the average bed level. c) The perturbation grows when transverse sediment transport is smaller than the difference between incoming and outgoing sediment transport. The middle grid cell will incise further, while the surrounding cells will accrete.

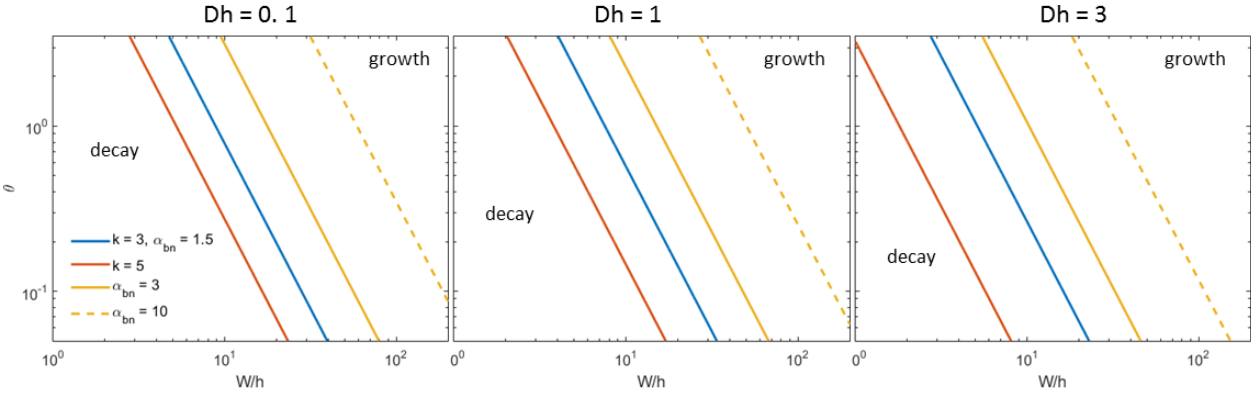


**Supplementary Figure 3:** The trend in equilibrium width-to-depth ratios with increasing sediment mobility, for three different depths of the initial perturbation ($dh$), resulting from the analytical model. Colors indicate the non-linearity of sediment transport (k). Solid lines indicate default slope effect ($\alpha_I$ = 1.5), dashed lines indicate an increased slope effect ($\alpha_I$ = 10). Width-to-depth ratios to the left of these lines will result in a decay of the initial perturbation, while ratios towards the right will result in a growth.

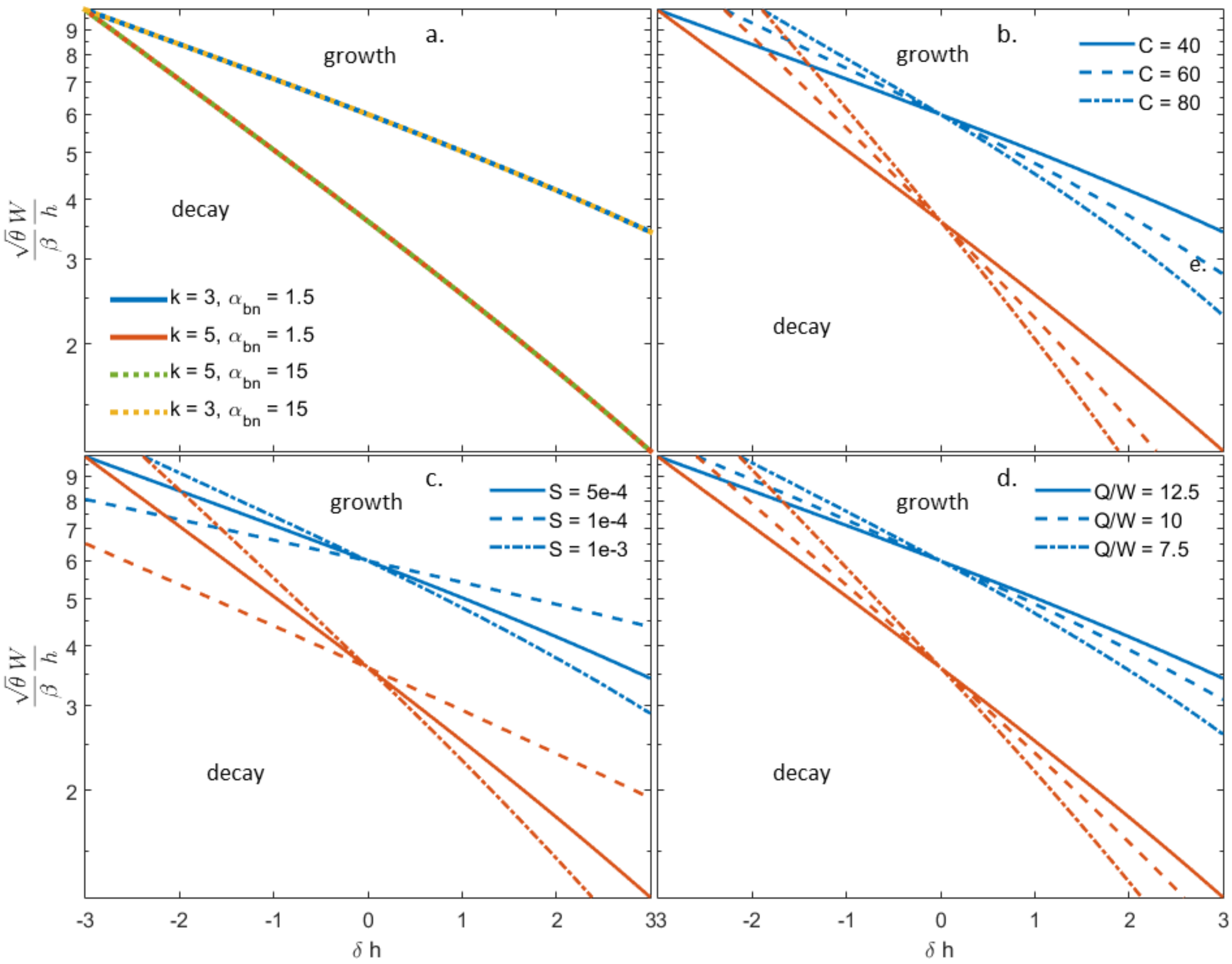


**Supplementary Figure 4:** Channelization factor resulting from the analytical model, plotted against the depth of the initial perturbation. Width-to-depth ratios lower than these lines will result in a decay of the initial perturbation, while higher ratios will result in a growth.

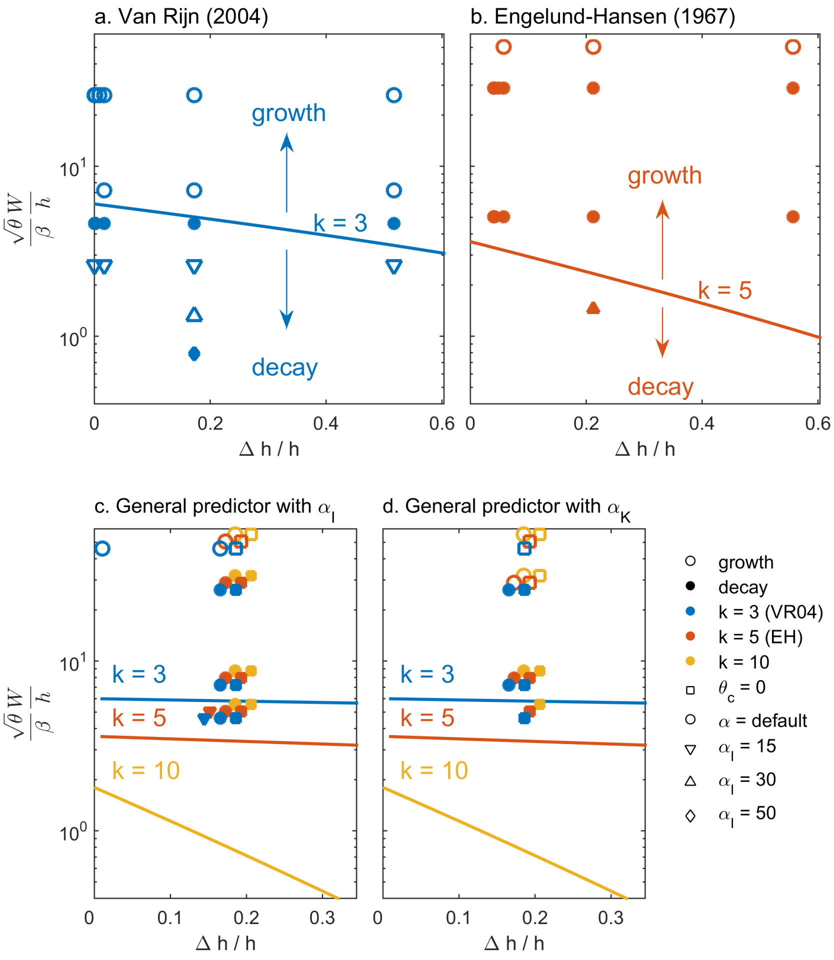


**Supplementary Figure 5:** Comparison between the behavior of the analytical model (lines) and the numerical channel (symbols) with (a) the VR sediment transport predictor, (b) the EH sediment transport predictor, (c) the general transport predictor with the IK slope parameterization, and (d) the general transport predictor with the KF slope parameterization. The analytical model predicts the width-to-depth ratio (W/h) for a certain non-linearity of sediment transport (power k on shear stress) at which incision and downslope sediment transport are in balance. The channelization factor ($W/h\sqrt{\theta}/\beta$) is plotted against the relative depth of the initial perturbation ($\Delta h/h$). The perturbation in the numerical models either grows (open symbol), or decays (filled symbol). Colors represent non-linearity of the sediment transport predictor and symbols represent the magnitude of the slope effect based on the IK slope parameterization, or in case of the general transport predictor the absence of a critical sediment mobility.

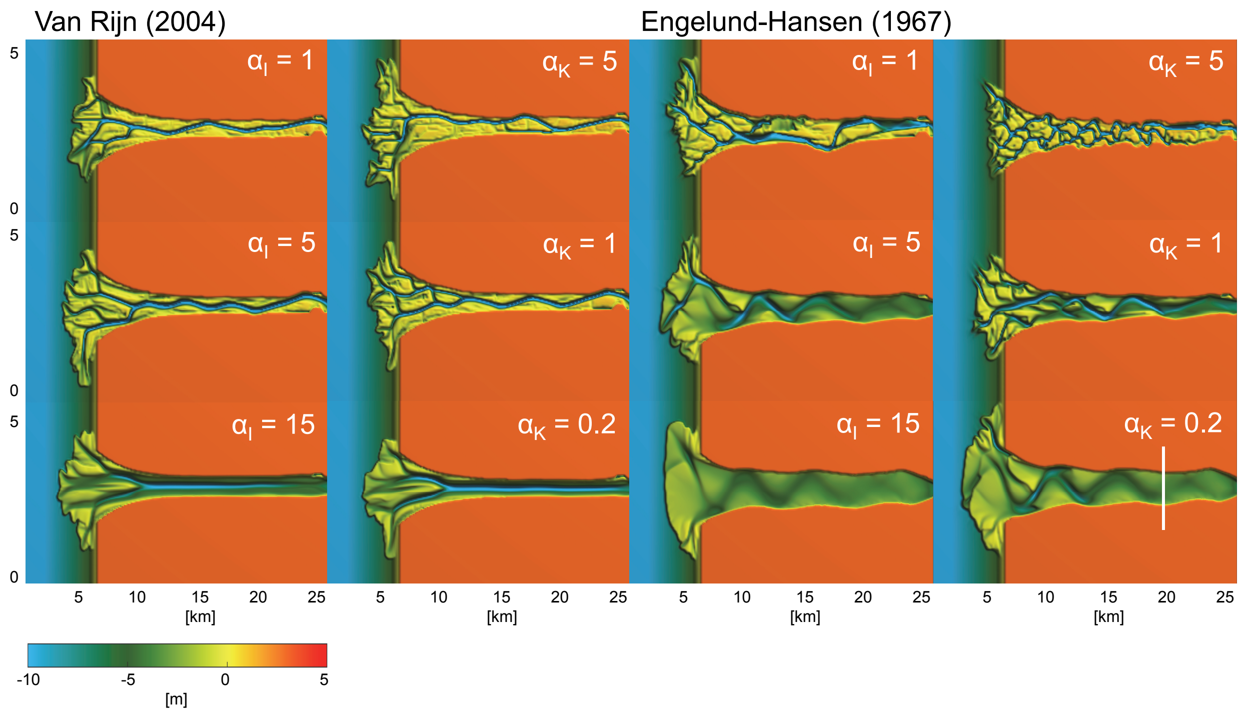


**Supplementary Figure 6:** Morphology of 12 delta model runs after 1000 years for combinations of slope effect and sediment transport predictors. Maps on the horizontal axis have an equal slope effect, with slope effect increasing downwards. The $\alpha_I$ is the input parameter of the method of Ikeda[2], while the $\alpha_K$ is the input parameter of the method of Koch and Flokstra[3]. The models in the first two columns were run with the VR sediment transport predictor, while the models in the last two columns were run with the EH predictor. The average sediment transport rates plotted in Supplementary Figure 10 were computed for all model runs over a cross-section at 20 km, represented by the white line in the bottom right panel.

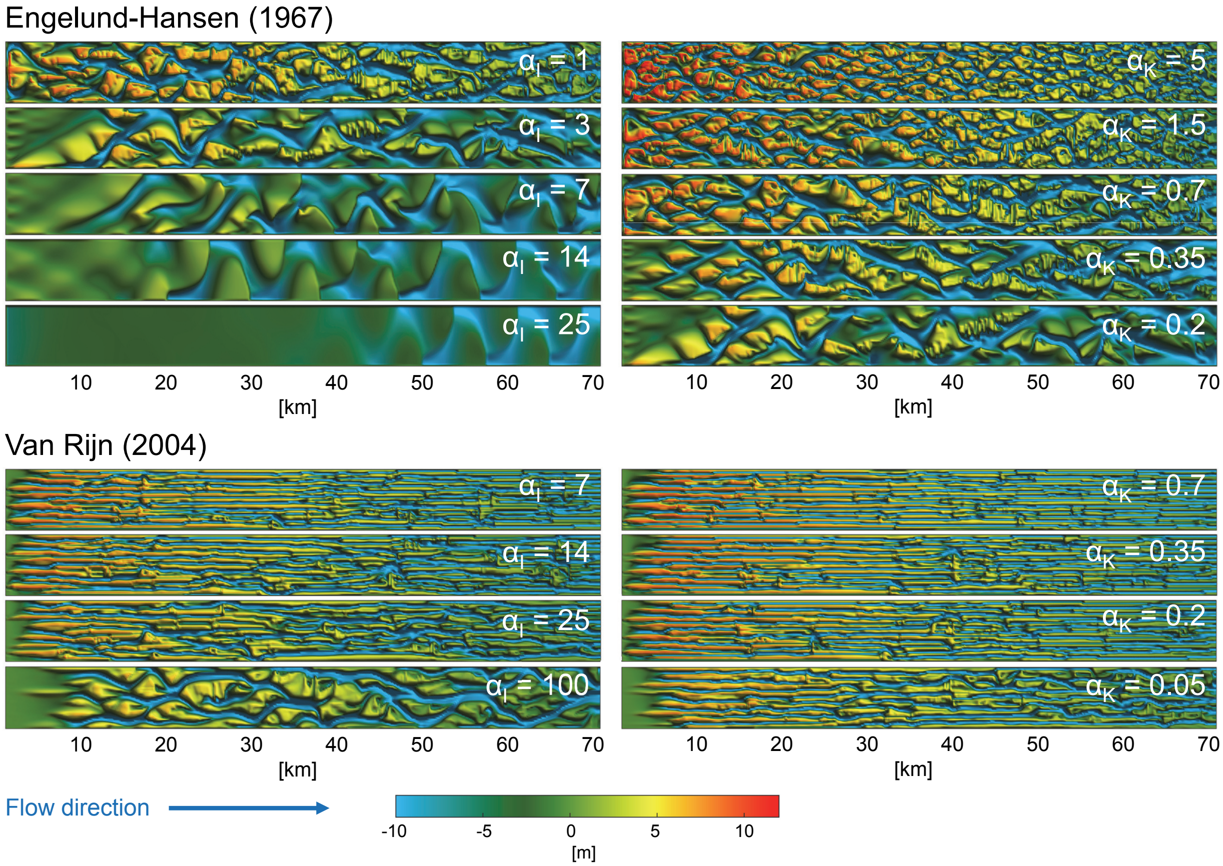


**Supplementary Figure 7:** Morphology of 18 braided river model runs for all combinations of slope effect and sediment transport predictors. Models on the horizontal axis have equal slope effect, which increases downwards for each transport predictor. The $\alpha_I$ is the input parameter of the method of Ikeda[2], while the $\alpha_K$ is the input parameter of the method of Koch and Flokstra[3], both with defaults of order 1.

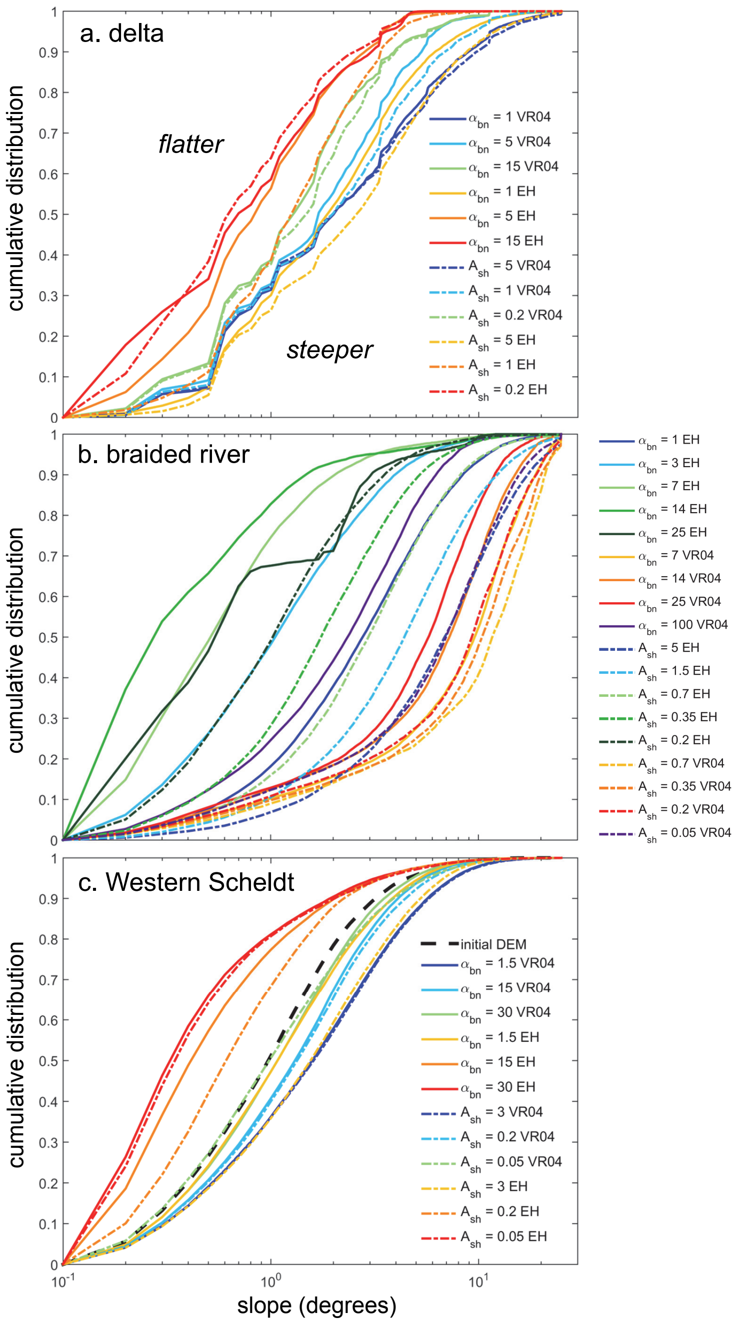


**Supplementary Figure 8:** Cumulative slope distributions of (a) the delta model runs, (b) the braided river model runs, and c) the Western Scheldt model runs. Solid lines are results with IK and dashed lines are results with KF. Colors indicate equal transverse sediment transport magnitudes and the same sediment transport predictor. The black dashed line in the Western Scheldt plot represents a measured bathymetry used as input.

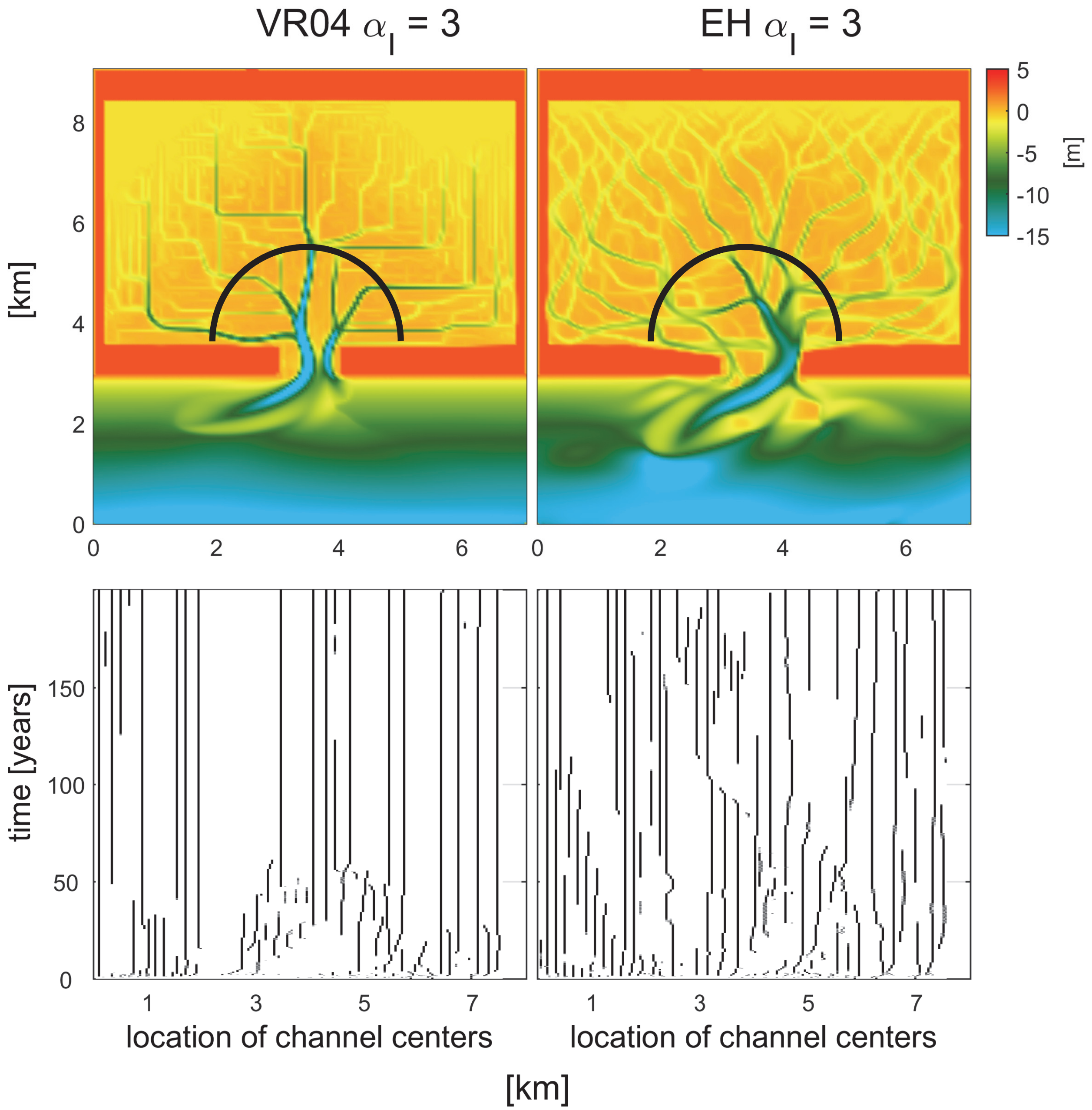


**Supplementary Figure 9:** Difference in morphology between the tidal basin model with either the sediment transport predictor of Van Rijn or Engelund-Hansen. The lower two panels show the locations of the channel centers over time at the cross-section that is indicated with the black circle in the upper two DEMs. The slope parameter is constant and modeled with the IK slope parameterization[2].

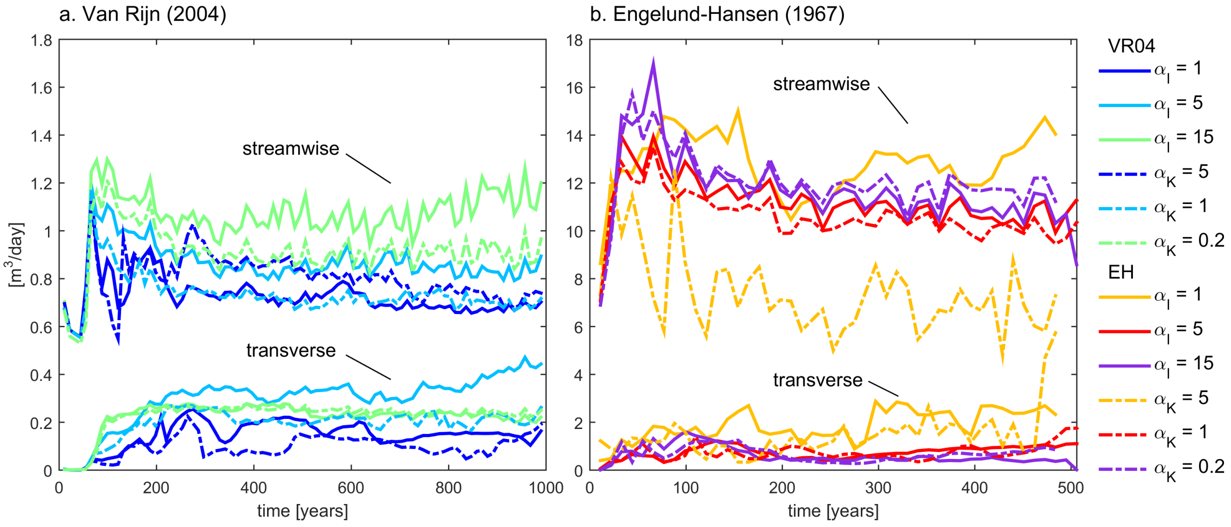


**Supplementary Figure 10:** Total sediment transport over a transect at km 20 in the river part of the delta model, integrated over the active channel width. (a) Streamwise and transverse sediment transport over time for all models with the VR sediment transport predictor, and (b) for all the model runs with the EH predictor. Note different vertical scales. Solid lines represent model runs with the IK method to calculate downslope sediment transport, while the dashed lines represent the models with the KF method. Lines with the same colors represent corresponding slope effects and sediment transport predictors.

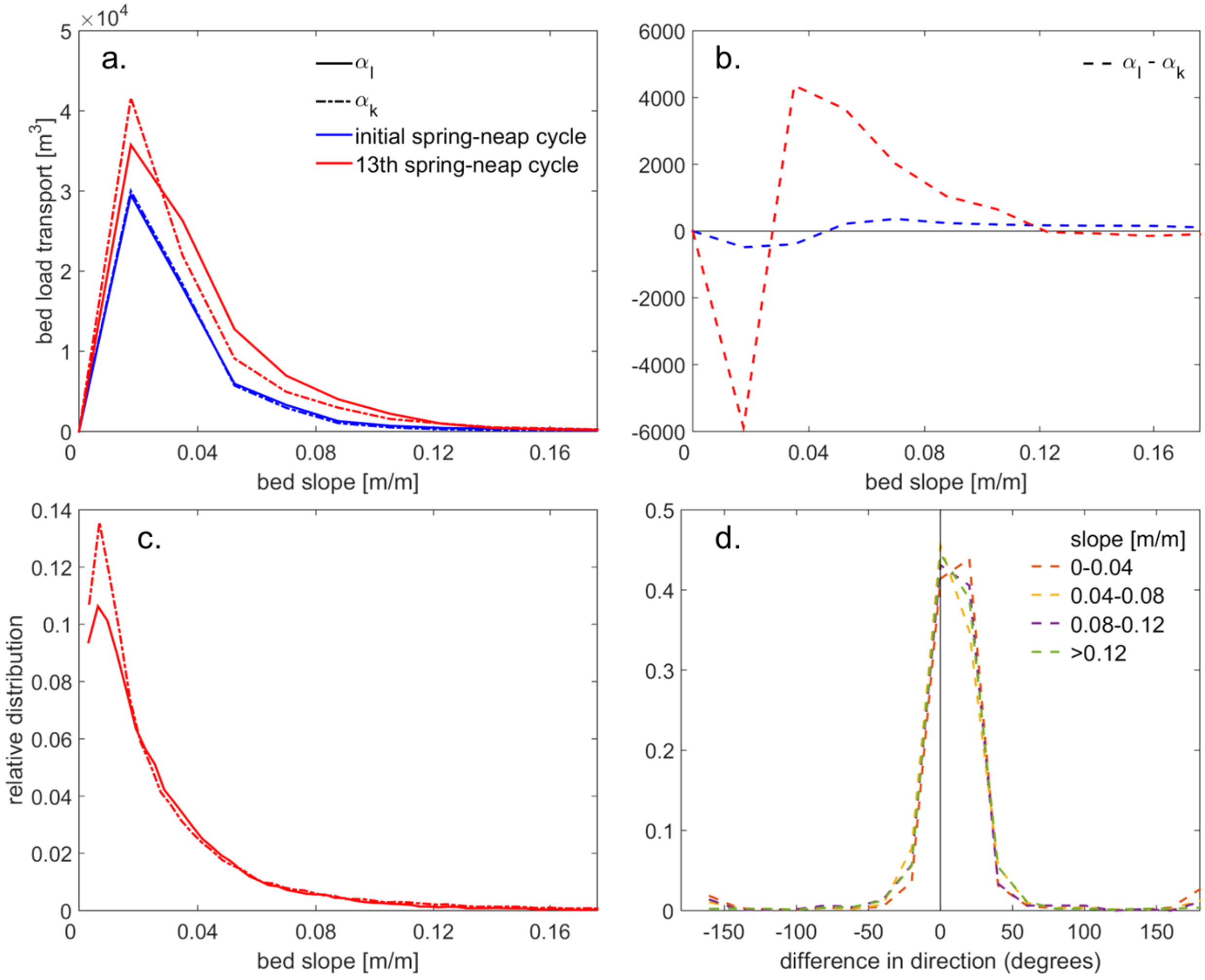


**Supplementary Figure 11:** (a) Total amount of sediment transported on specific bed slopes for the Western Scheldt models with different slope parameterizations, at the beginning and at the end of the model run. (b) The difference in sediment transport between both slope parameterizations on specific bed slopes. (c) relative distributions of bed slopes in both Western Scheldt models. (d) Difference in direction of sediment transport between the models with different slope parameterizations. This distribution shows the relative abundance of these differences for all grid cells in the model.

## Supplementary Tables

Supplementary Table 1 shows the results of the literature inventory in typical geomorphology journals. For each study, the morphodynamic model and the magnitude of the slope factor is indicated, and whether this study mentions or discusses the effect on morphology of the slope parameter (yes = 1, no = 0). The environment that is modelled can be erosional (1), depending on a large-scale balance between erosion and deposition (2), or depositional (3). Models are included in this literature study when they either have an upstream river boundary (1), a river boundary as well as a downstream tidal boundary (2), or only a tidal boundary (3). Lastly, it is noted whether the model considers suspension (1) or treats all sediment transport as bedload (0).

**Supplementary Table 1:** Results of the literature inventory of slope effects in morphodynamic models described in the main text

| Paper | model | slope effect | mention? yes/no (1/0) | discuss? yes/no (1/0) | environment e/b/d (1/2/3) | boundary r,r+t,t (1/2/3) | suspension? yes/no (1/0) |
|---|---|---|---|---|---|---|---|
| Yuill2016[4] | Delft3D | 1,5 | 0 | 0 | 1 | 1 | 1 |
| Asahi2013[5] | Nays2D | 1,5 | 0 | 0 | 1 | 1 | 0 |
| vdWegen2008[6] | Delft3D | 1,5 | 1 | 1 | 1 | 2 | 0 |
| Tran2012[7] | Delft3D | 1,5 | 0 | 0 | 1 | 3 | 1 |
| Tuan2008[8] | MOGEC | 2 | 1 | 0 | 1 | 1 | 1 |
| Dassa2009[9] | Delft3D | 5 | 1 | 1 | 1 | 3 | 0 |
| Marciano2005[10] | Delft3D | 5 | 1 | 0 | 1 | 3 | 0 |
| Zhou2014[11] | Delft3D | 1,5 | 1 | 1 | 1 | 3 | 0 |
| Schuurman2013[12] | Delft3D | 15 | 1 | 1 | 2 | 1 | 0 |
| Oorschot2016[13] | Delft3D | 15 | 1 | 0 | 2 | 1 | 0 |

| | | | | | | | |
|---|---|---|---|---|---|---|---|
| Caldwell2014[14] | Delft3D | 1,5 | 1 | 0 | 3 | 1 | 1 |
| Matsubara2014[15] | Delft3D | 4,4 | 1 | 0 | 1 | 1 | 1 |
| Schuurman2015b[16] | Delft3D | 7 | 1 | 0 | 2 | 1 | 0 |
| Stecca2017[17] | Delft3D | 1,43 | 1 | 0 | 1 | 1 | 0 |
| Williams2016[18] | Delft3D | 1,5 | 1 | 1 | 1 | 1 | 0 |
| Edmonds2008[19] | Delft3D | 1,5 | 1 | 1 | 3 | 1 | 1 |
| Schuurman2016[20] | Delft3D/ Nays2D | 6 | 1 | 0 | 2 | 1 | 0 |
| Jimenez2016[21] | Delft3D | 1,5 | 0 | 0 | 3 | 1 | 1 |
| Nicholas2013b[22] | Delft3D | 25 | 1 | 1 | 2 | 1 | 1 |
| Xie2009[23] | Delft3D | 1,5 | 0 | 0 | 1 | 1 | 1 |
| vdWegen2012[24] | Delft3D | 10 | 1 | 1 | 2 | 2 | 0 |
| Nnafie2018[25] | Delft3D | 2 | 1 | 0 | 1 | 2 | 0 |
| Leonardi2013[26] | Delft3D | 1,5 | 1 | 1 | 3 | 2 | 1 |
| vdWegen2014[27] | Delft3D | 100 | 1 | 0 | 2 | 2 | 1 |
| VanDijk2018b[28] | Delft3D | 30 | 1 | 0 | 2 | 2 | 1 |
| Nienhuis2016[29] | Delft3D | 1,5 | 0 | 0 | 3 | 1 | 1 |
| Kleinhans2010b[30] | Delft3D | 1,5 | 0 | 0 | 3 | 1 | 1 |
| Edmonds2007[31] | Delft3D | 1,5 | 1 | 0 | 3 | 1 | 1 |
| VanDijk2014[32] | Delft3D | 7 | 1 | 1 | 1 | 1 | 0 |
| Schuurman2018[33] | Delft3D | 25 | 1 | 1 | 2 | 1 | 0 |
| VanMaren2009[34] | Delft3D | - | 0 | 0 | 2 | 1 | 1 |
| Vargas2018[35] | Delft3D | - | 0 | 0 | 2 | 1 | 0 |
| Liedermann2018[36] | iSed | - | 0 | 0 | 2 | 1 | 1 |
| Martinez2018[37] | Delft3D | - | 0 | 0 | 2 | 1 | 0 |
| Mariotti2013[38] | Delft3D | - | 0 | 0 | 3 | 1 | 1 |
| Canestrelli2014[39] | Delft3D | - | 0 | 0 | 3 | 1 | 1 |
| Yuill2016b[40] | Delft3D | - | 0 | 0 | 3 | 1 | 1 |
| Rousseau2017[41] | Telemac | - | 0 | 0 | 1 | 1 | 0 |
| Norman2017[42] | Nays2D | - | 0 | 0 | 1 | 1 | 1 |
| French2010[43] | Telemac | - | 0 | 0 | 2 | 2 | 1 |

| | | | | | | | |
|---|---|---|---|---|---|---|---|
| French2003[44] | Telemac | - | 0 | 0 | 2 | 2 | 1 |
| Son2011[45] | Delft3D | - | 0 | 0 | 1 | 2 | 1 |
| Xie2017[46] | Delft3D | - | 0 | 0 | 2 | 2 | 1 |
| vdWegen2011[47] | Delft3D | - | 0 | 0 | 2 | 2 | 1 |
| Ralston2017[48] | ROMS +CSTMS | - | 0 | 0 | 3 | 2 | 1 |
| Ganju2009[49] | ROMS +CSTMS | - | 0 | 0 | 2 | 2 | 1 |
| Luan2017[50] | Delft3D | - | 0 | 0 | 2 | 2 | 1 |
| Davies2017[51] | Telemac | - | 0 | 0 | 2 | 2 | 1 |
| Hibma2004[52] | Delft3D | - | 0 | 0 | 2 | 2 | 1 |
| Guo2015[53] | Delft3D | - | 0 | 0 | 2 | 2 | 0 |
| Mariotti2017[54] | Delft3D | - | 0 | 0 | 1 | 3 | 1 |
| Schwarz2014[55] | Delft3D | - | 0 | 0 | 1 | 3 | 1 |
| Horstman2015[56] | Delft3D | - | 0 | 0 | 1 | 3 | 1 |
| Dissa2012[57] | Delft3D | - | 0 | 0 | 1 | 3 | 1 |
| Hu2018[58] | Delft3D | - | 0 | 0 | 1 | 3 | 1 |
| Rossi2016[59] | Delft3D | 1,5 | 0 | 0 | 3 | 1 | 1 |
| Hajek2014[60] | Delft3D | 1,5 | 0 | 0 | 3 | 1 | 1 |
| Geleynse2010[61] | Delft3D | 1,5 | 0 | 0 | 3 | 1 | 0 |
| vdLageweg2018[62] | Delft3D | 1,5 | 0 | 0 | 3 | 2 | 0 |
| Hibma2003[63] | Delft3D | 1 | 1 | 0 | 2 | 2 | 0 |
| Lotsari2014[64] | TUFLOW | 2 | 1 | 0 | 2 | 1 | 1 |
| Braat2017[65] | Delft3D | 25 | 1 | 1 | 2 | 2 | 0 |
| vdVegt2016[66] | Delft3D | 1,5 | 0 | 0 | 3 | 1 | 1 |
| Edmonds2010[67] | Delft3D | 1,5 | 0 | 0 | 3 | 1 | 1 |
| Temmerman2007[68] | Delft3D | 1,5 | 0 | 0 | 1 | 3 | 1 |
| Ridderinkhof2014[69] | Delft3D | 20 | 1 | 0 | 1 | 3 | 1 |
| VanLeeuwen2003[70] | Delft3D | 1,5 | 0 | 0 | 1 | 3 | 0 |
| Sanyal2017[71] | Delft3D | 1,5 | 0 | 0 | 1 | 1 | 0 |

## Supplementary Notes

**Supplementary Note 1** To understand the implementation of the bed slope effect in Delft3D and how it interacts with sediment transport predictors, we first explain the calculation of streamwise sediment transport. In this study, we focus on the difference between the Van Rijn[72] and Engelund-Hansen[73] sediment transport predictor and their influence on the balance of incision and downslope sediment transport. Furthermore, we briefly compare these results with a general sediment transport predictor, where the non-linearity of sediment transport and the addition of a critical shear stress can be specified by the user. Henceforth, we refer to the predictor of Van Rijn as VR, and to the predictor of Engelund-Hansen as EH.

VR makes a distinction between bed load and suspended load transport, by imposing a reference height, below which sediment transport is treated as bed load and everything above this height is treated as suspended load. Gravity only acts on the bed load, which is calculated as follows:

$$q_b = 0.5 \rho_s d_{50} D_*^{-0.3} \left(\frac{\tau}{\rho}\right)^{0.5} \frac{\tau - \tau_{cr}}{\tau_{cr}} \tag{1}$$

where $q_b$ = bed load sediment transport rate per meter width [m$^2$s$^{-1}$], $\rho_s$ = sediment density [kg m$^{-3}$], $\rho_w$ = water density [kg m$^{-3}$], $D_{50}$ = median grainsize [m], $D_*$ = dimensionless particle size, $\tau$ = shear stress [N m$^{-2}$], $\rho$ = density [kg m$^{-3}$], $\tau_{cr}$ = critical shear stress based on the Shields criterion [N m$^{-2}$]. As a result, the sediment transport rate is related to flow velocity to the power of 3, which determines the non-linearity of the sediment transport predictor. However, since this

predictor also includes a critical flow velocity, the relation between flow velocity and sediment transport will be more non-linear near the beginning of motion.

EH is a total load predictor ($q_t$), and unlike VR, it does not include a critical velocity or critical shear stress:

$$q_t = \frac{0.005\alpha u^5}{\sqrt{g}C^3\frac{\rho_s-\rho_w}{\rho_w}D_{50}} \tag{2}$$

where $\alpha$ = a calibration coefficient in the order of 1. Here, the sediment transport rate is related to flow velocity to the power of 5.

The general sediment transport predictor in Delft3D is based on the predictor of Meyer-Peter Mueller[74]:

$$q_t = \alpha D_{50}\sqrt{\frac{\rho_s-\rho_w}{\rho_w}gD_{50}}\theta^b(\theta-\theta_c)^c \tag{3}$$

where b and c are user defined parameters, which determine the non-linearity of the sediment transport predictor and the addition of a critical sediment mobility. The sediment mobility $\theta$, a dimensionless form of the bed shear stress, reads:

$$\theta = \frac{u^2}{C^2\frac{\rho_s-\rho_w}{\rho_w}D_{50}} \tag{4}$$

When the magnitude of the bed load or total load sediment transport is calculated parallel to the flow velocity, the direction and magnitude of the transport vector is adjusted for bed slopes. For transverse slopes, the two commonly used parameterizations are the predictor of Koch and Flokstra (KF)[3] (ISlope = 3) and Ikeda (IK)[2] (ISlope = 2). The main difference between both options is in the calculation of the transport vector (Fig. 2 in main text). For KF the direction of sediment transport is corrected for transverse gradients by rotating the transport vector based on the user-defined factors $\alpha_K$ and $\beta_K$:

$$tan(\psi) = \frac{1}{\alpha_K \theta^{\beta_K}} \frac{\partial z_b}{\partial y} \tag{5}$$

For IK an additional transport vector is calculated perpendicular to the flow direction, based on the input parameter $\alpha_I$:

$$q_n = q_s \alpha_I \sqrt{\frac{\theta_c}{\theta}} \frac{\partial z_b}{\partial y} \tag{6}$$

where $q$ = sediment transport load [m$^2$s$^{-1}$] in the streamwise (s) or transverse (n) direction, and $\frac{\partial z_b}{\partial y}$ = transverse slope [m m$^{-1}$].

As a result, the IK method increases the direction and total magnitude of sediment transport when a transverse slope is present, while for KF only the direction is changed. Another difference is that the IK method uses a critical shear stress, which is absent in the KF method. The default

value of $\alpha_I$ in Delft3D is set to 1.5, while the parameter $\alpha_K$ is not defined in the model, but should be 1.5 according to Koch and Flokstra[3]. The method of calculating the sediment transport vector in both slope options therefore has major implications for calibrating models with the transverse slope parameter. By increasing the $\alpha_I$ in the IK method by a factor of ten for example, the amount of downslope sediment transport is also increased by a factor of ten, which increases the total sediment transport significantly (Fig. 2 in main text). With the KF method sediment transport is not increased, but here, decreasing the $\alpha_K$ to values reported in literature[12,13,65] could easily result in more downslope sediment transport than streamwise sediment transport.

It is possible to compare the effect on resulting morphology of using different slope predictors by requiring either the magnitude or the direction of transverse sediment transport to be equal. When assuming an equal magnitude, the method of KF needs to be corrected for a given slope and sediment mobility. Using Equation 5 and Equation 6 with a $\beta_K$ of 0.5 it follows that:

$$\alpha_I = \left( \alpha_K \sqrt{\theta_c + \frac{\theta_c}{\alpha_K^2 \theta} \left( \frac{dz}{dy} \right)^2} \right)^{-1} \tag{7}$$

The resulting relation between $\alpha_I$ and $\alpha_K$ is plotted in Supplementary Figure. 1 for four combinations of transverse slope and sediment mobility. When assuming equal direction of sediment transport, it follows that:

$$\alpha_I = \left( \alpha_K \sqrt{\theta_c} \right)^{-1} \tag{8}$$

which is shown as the linear solution in Supplementary Figure 1.

**Supplementary Note 2** To help identify the cause of the overdeepening of channels in numerical models, we compare the balance between incision and transverse sediment transport in a straight river channel in Delft3D with an analytical model of a channel cross-section with the same characteristics. Since we only consider a cross-section, the streamwise sediment transport is in balance with the constant flow conditions and the model does not account for deposition along a river reach. The analytical model consists of three grid cells in cross-section, with an initial bed level difference between the middle cell and the surrounding cells, representing a disturbance that either decays or grows by incising further (Supplementary Figure 2). The aim of this model is to find the equilibrium width-to-depth ratio at which incision is equal to transverse sediment transport, and how this ratio depends on flow conditions, sediment transport processes, and size of the disturbance.

The model first calculates upstream flow characteristics and corresponding sediment transport rate based on the input parameters, which are a constant Chezy coefficient for friction ($C$), channel slope ($S$), grain size ($D_{50}$), the non-linearity of the sediment transport predictor ($k$), and a height difference ($dh$). We assume a constant specific discharge such that the relation between channel width ($W$) and discharge ($Q$) is linear:

$$Q = aW \tag{9}$$

The upstream flow velocity ($u_i$) and water depth ($h_i$) are calculated by iteration, using the

following equations for flow velocity:

$$u_i = C\sqrt{h_i S} \tag{10}$$

$$u_i = \frac{a}{h_i} \tag{11}$$

The upstream sediment transport rate ($q_i$) is based on the same general sediment transport predictor as in Delft3D:

$$q_i = \alpha D_{50}\sqrt{\frac{\rho_s - \rho_w}{\rho_w} g D_{50}}\theta_i^{\frac{k}{2}} \tag{12}$$

Then, flow characteristics and sediment transport fluxes are calculated for the cross-section under consideration, based on the height difference between the middle grid cell ($h_2$) and the outer two grid cells ($h_1$, $h_3$) (Supplementary Figure 2a). It is assumed that the average water depth at the cross-section is equal to the initial water depth, which leads to:

$$h_1 = h_3 = h_i - 0.5dh \tag{13}$$

$$h_2 = h_i + dh \tag{14}$$

The sediment transport rate for each cell is then calculated with Equations 4, 10 and 12, but with the specific water depths. The sediment transport rate towards the middle cell as a result of the transverse slope ($q_n$) is based on the method of Ikeda[2]:

$$q_n = q_1 \frac{\beta}{\sqrt{\theta}} \frac{1.5dh}{\frac{W}{3}} \tag{15}$$

where $\beta$ = transverse slope parameter, which is based on $\alpha_I$ from Equation 6. The transverse slope is defined as the height difference between two cells divided by the width of one grid cell, which is the same method as in Delft3D.

A balance between incision and downslope sediment transport is assumed when the difference between the upstream sediment transport and the sediment transport rate for the middle grid cell is equal to the total downslope sediment transport:

$$2q_n = q_2 - q_i \tag{16}$$

When the transverse sediment flux is larger, there is sedimentation and the perturbation will likely decay (Supplementary Figure 2b), while when the transverse sediment flux is smaller, the grid cell is incised and the perturbation will grow (Supplementary Figure 2c). Using Equations 4, 10 and 12 it follows that:

$$W_{eq} = \frac{h_1^{k/2}}{h_2^{k/2} - h_i^{k/2}} \frac{9\beta dh}{\sqrt{\theta}} \tag{17}$$

where $W_{eq}$ = width of the channel when incision is equal to the transverse sediment transport. The equilibrium width-to-depth ratio is now a function of the size of the disturbance, sediment mobility and the non-linearity of sediment transport. All other parameters influence this equilibrium by changing the sediment mobility. In further analyses we first assume a constant channel slope of 0.5 mm m$^{-1}$, a Chezy coefficient of 40 $\sqrt{m}/s^{-1}$, a ratio between channel width and discharge of 12.5, and a grain size of 0.5 $mm$.

With increasing sediment mobility, the equilibrium width-to-depth ratio decreases exponentially (Supplementary Figure 3), which means that at higher sediment mobility a channel is more likely to incise. A higher non-linearity of the transport predictor causes a higher sediment transport rate, and therefore results in more incision and a lower equilibrium width-to-depth ratio at any sediment mobility. Increasing the transverse slope parameter has the opposite effect, since more sediment is transported downslope which counteracts incision. Increasing the depth of the initial perturbation also decreases the equilibrium width (Supplementary Figure 3), since deeper channels attract more flow and therefore need more downslope sediment transport to counteract this. However, this influence is less than changing the bed slope effect or the non-linearity.

To be able to show the effects of height of the perturbation and the other parameters that influence sediment mobility, the width-to-depth ratio is multiplied by the square root of the sed-

iment mobility divided by the slope parameter, which is the ratio that describes the slopes of the graphs in Supplementary Figure 3. We call the resulting parameter the channelization factor, since it describes the balance between the tendency to enhance perturbations determined by the width-to-depth ratio, and the bed slope effect that counteracts incision. This balance thereby controls the formation of channels. As a result, Supplementary Figure 4a shows how models with varying slope effect and sediment mobility collapse when plotting this factor against height of the perturbation. Again, a higher non-linearity of sediment transport results in a growth of the perturbation at lower width-to-depth ratios. Higher Chezy values, and thus lower friction, also results in a growth of the perturbation at lower width-to-depth ratios when increasing the depth of the perturbation, but less dramatically. However, negative perturbations, i.e. when the middle grid cell is higher than the surrounding cells, need higher width-to-depth ratios for the perturbation to grow. Increasing the channel slope or decreasing the ratio between discharge and channel width shows the same trend.

Since the analytical model identifies the equilibrium channelization factor, perturbations in numerical models plotted below this line should theoretically decay, while models plotted above the line should have growing perturbations (Supplementary Figure 5). With the default value for the slope effect ($\alpha_I$ = 1.5), the VR models corresponded reasonably well with the analytical model, since the transition from a dampened system towards a channel where the perturbation grows is around the theoretical equilibrium line (Supplementary Figure 5a). There was no effect of the depth of the initial perturbation in the numerical model. However, with increased slope effect, the numerical models significantly deviated from the analytical model. Here, the numerical model with wider channels required a disproportionately larger slope effect to dampen the initial

perturbation (more than 30 times higher than the default factor as opposed to 4 times the default in the analytical model). On the other hand, the initial perturbation in models with the models with the EH predictor immediately decayed (Supplementary Figure 5b), until the channel has a width-to-depth ratio around 36, which is more than 15 times higher than the theoretical model. This behavior was very similar to that of the models with the general predictor (Supplementary Figure 5c,d). Even when sediment transport is related to flow velocity to the power of 10, perturbations did not start to grow at a lower width-to-depth-ratio, while this was expected based on the analytical model. The two slope parameterizations differed only slightly and removing the critical sediment mobility from the generic transport predictor had no effect on equilibrium morphology. These results demonstrate a stronger tendency to incise in the numerical model with VR than expected from theory, and a weaker tendency to incise in numerical models with EH.

**Supplementary Note 3** The fifth set of models is a detailed case study of the topographically forced Western Scheldt estuary in the Netherlands, to test the sensitivity of a calibrated model with two different slope parameterizations in comparison with measured bathymetry. This topographic forcing is typical for many natural and engineered systems and is important because it limits free bar and pattern formation, rendering models less sensitive in large-scale pattern to chosen parameterizations. Here, we focused on differences in local sediment transport dynamics in two model runs with different slope predictors that showed the same large-scale morphology in view of the need to predict sediment transport rates for fairway maintenance dredging. After 10 years of morphological development, these models reproduced the cumulative slope distributions that were closest to the actual morphology of the Western Scheldt that was used as input (Supple-

mentary Figure 8c). The models had a strong slope effect, namely an $\alpha_I$ of 30 and an $\alpha_K$ of 0.05, which again shows that a higher than physical slope effect is needed when calibrating the Western Scheldt model on existing morphology.

While large-scale morphology is similar between both models after ten years (Supplementary Figure 8d), the dynamics differ in local sediment transport. The model with the IK method has higher bed load transport rates on steeper slopes, while the model with the KF method has higher transport rates on lower slopes (Supplementary Figure 11). Furthermore, there is a significant difference in direction of the transport vectors in more than half of all grid cells in the model (Supplementary Figure 11d), which is independent of slope. These differences in direction and magnitude imply locally channels can be orientated differently and location and speed of bank erosion will differ. For fairway maintenance dredging this means that predicted time scales can significantly differ when models are calibrated with a different slope parametrization on the same measured morphology.

**Supplementary References**


1. Baar, A. W., de Smit, J., Uijttewaal, W. S. & Kleinhans, M. G. Sediment transport of fine sand to fine gravel on transverse bed slopes in rotating annular flume experiments. *Water Resources Research* **54**, 19–45 (2018).

2. Ikeda, S. Lateral bed-load transport on side slopes - closure. *Journal of Hydraulic Engineering* **110**, 200–203 (1984).

3. Koch, F. & Flokstra, C. Bed level computations for curved alluvial channels. In *19th International association for hydraulic research congress* (1981).

4. Yuill, B. T., Gaweesh, A., Allison, M. A. & Meselhe, E. A. Morphodynamic evolution of a lower mississippi river channel bar after sand mining. *Earth Surface Processes and Landforms* **41**, 526–542 (2016).

5. Asahi, K., Shimizu, Y., Nelson, J. & Parker, G. Numerical simulation of river meandering with self-evolving banks. *Journal of Geophysical Research: Earth Surface* **118**, 2208–2229 (2013).

6. Van Der Wegen, M. & Roelvink, J. A. Long-term morphodynamic evolution of a tidal embayment using a two-dimensional, process-based model. *Journal of Geophysical Research: Oceans* **113**, 1–23 (2008).

7. Tran, T.-t., Kreeke, J. V. D., Stive, M. J. F. & Walstra, D.-j. R. Cross-sectional stability of tidal inlets : A comparison between numerical and empirical approaches. *Coastal Engineering* **60**, 21–29 (2012). URL http://dx.doi.org/10.1016/j.coastaleng.2011.08.005.

8. Tuan, T. Q., Stive, M. J., Verhagen, H. J. & Visser, P. J. Process-based modeling of the overflow-induced growth of erosional channels. *Coastal Engineering* **55**, 468–483 (2008).

9. Dissanayake, D., Roelvink, J. & Van der Wegen, M. Modelled channel patterns in a schematized tidal inlet. *Coastal Engineering* **56**, 1069–1083 (2009).

10. Marciano, R., Wang, Z. B., Hibma, A., Vriend, H. J. D. & Defina, A. Modeling of channel patterns in short tidal basins. *Journal of Geophysical Research* **110**, 1–13 (2005).

11. Zhou, Z. *et al.* A comparative study of physical and numerical modeling of tidal network ontogeny. *Journal of Geophysical Research: Earth Surface* **119**, 892–912 (2014).

12. Schuurman, F., Marra, W. a. & Kleinhans, M. G. Physics-based modeling of large braided sand-bed rivers: Bar pattern formation, dynamics, and sensitivity. *Journal of Geophysical Research: Earth Surface* **118**, 2509–2527 (2013).

13. Oorschot, M. v., Kleinhans, M., Geerling, G. & Middelkoop, H. Distinct patterns of interaction between vegetation and morphodynamics. *Earth Surface Processes and Landforms* **41**, 791–808 (2016).

14. Caldwell, R. L. & Edmonds, D. A. Journal of Geophysical Research : Earth Surface. *Journal of Geophysical Research: Earth Surface* **119**, 961–982 (2014).

15. Matsubara, Y. & Howard, A. D. Modeling planform evolution of a mud-dominated meandering river: Quinn river, nevada, usa. *Earth Surface Processes and Landforms* **39**, 1365–1377 (2014).

16. Schuurman, F. & Kleinhans, M. G. Bar dynamics and bifurcation evolution in a modelled braided sand-bed river. *Earth surface processes and landforms* **40**, 1318–1333 (2015).

17. Stecca, G., Measures, R. & Hicks, D. A framework for the analysis of noncohesive bank erosion algorithms in morphodynamic modeling. *Water Resources Research* **53**, 6663–6686 (2017).

18. Williams, R., Measures, R., Hicks, D. & Brasington, J. Assessment of a numerical model to reproduce event-scale erosion and deposition distributions in a braided river. *Water resources research* **52**, 6621–6642 (2016).

19. Edmonds, D. & Slingerland, R. Stability of delta distributary networks and their bifurcations. *Water Resources Research* **44** (2008).

20. Schuurman, F., Shimizu, Y., Iwasaki, T. & Kleinhans, M. Dynamic meandering in response to upstream perturbations and floodplain formation. *Geomorphology* **253**, 94–109 (2016).

21. Jiménez-Robles, A., Ortega-Sánchez, M. & Losada, M. Effects of basin bottom slope on jet hydrodynamics and river mouth bar formation. *Journal of Geophysical Research: Earth Surface* **121**, 1110–1133 (2016).

22. Nicholas, A., Ashworth, P., Sambrook Smith, G. & Sandbach, S. Numerical simulation of bar and island morphodynamics in anabranching megarivers. *Journal of Geophysical Research: Earth Surface* **118**, 2019–2044 (2013).

23. Xie, D., Wang, Z., Gao, S. & De Vriend, H. Modeling the tidal channel morphodynamics in a macro-tidal embayment, hangzhou bay, china. *Continental Shelf Research* **29**, 1757–1767 (2009).

24. van der Wegen, M. & Roelvink, J. A. Reproduction of estuarine bathymetry by means of a process-based model: Western Scheldt case study, the Netherlands. *Geomorphology* **179**, 152–167 (2012).

25. Nnafie, A., Van Oyen, T., De Maerschalck, B., van der Vegt, M. & Wegen, M. v. d. Estuarine channel evolution in response to closure of secondary basins: An observational and morphodynamic modeling study of the western scheldt estuary. *Journal of Geophysical Research: Earth Surface* **123**, 167–186 (2018).

26. Leonardi, N., Canestrelli, A., Sun, T. & Fagherazzi, S. Effect of tides on mouth bar morphology and hydrodynamics. *Journal of Geophysical Research: Oceans* **118**, 4169–4183 (2013).

27. Van der Wegen, M. & Jaffe, B. Processes governing decadal-scale depositional narrowing of the major tidal channel in san pablo bay, california, usa. *Journal of Geophysical Research: Earth Surface* **119**, 1136–1154 (2014). URL

28. van Dijk, W., Hiatt, M., van der Werf, J. & Kleinhans, M. G. Effect of perturbations by shoal margin collapses on the morphodynamics of a sandy estuary. *Earth Surface Processes and Landforms* (2018).

29. Nienhuis, J. H., Ashton, A. D., Nardin, W., Fagherazzi, S. & Giosan, L. Alongshore sediment bypassing as a control on river mouth morphodynamics. *Journal of Geophysical Research: Earth Surface* **121**, 664–683 (2016).

30. Kleinhans, M. G., Weerts, H. J. & Cohen, K. M. Avulsion in action: reconstruction and modelling sedimentation pace and upstream flood water levels following a medieval tidal-river diversion catastrophe (biesbosch, the netherlands, 1421–1750 ad). *Geomorphology* **118**, 65–79 (2010).

31. Edmonds, D. & Slingerland, R. Mechanics of river mouth bar formation: Implications for the morphodynamics of delta distributary networks. *Journal of Geophysical Research: Earth Surface* **112** (2007).

32. Van Dijk, W. M., Schuurman, F., Van de Lageweg, W. I. & Kleinhans, M. G. Bifurcation instability and chute cutoff development in meandering gravel-bed rivers. *Geomorphology* **213**, 277–291 (2014).

33. Schuurman, F. *et al.* Response of braiding channel morphodynamics to peak discharge changes in the upper yellow river. *Earth Surface Processes and Landforms* (2018).

34. Van Maren, D., Winterwerp, J., Wu, B. & Zhou, J. Modelling hyperconcentrated flow in the yellow river. *Earth Surface Processes and Landforms* **34**, 596–612 (2009).

35. Vargas-Luna, A. *et al.* Morphodynamic effects of riparian vegetation growth after stream restoration. *Earth Surface Processes and Landforms* (2018).

36. Liedermann, M., Gmeiner, P., Kreisler, A., Tritthart, M. & Habersack, H. Insights into bed-load transport processes of a large regulated gravel-bed river. *Earth Surface Processes and Landforms* **43**, 514–523 (2018).

37. Martínez-Fernández, V., Van Oorschot, M., De Smit, J., González del Tánago, M. & Buijse, A. D. Modelling feedbacks between geomorphological and riparian vegetation responses under climate change in a mediterranean context. *Earth Surface Processes and Landforms* (2018).

38. Mariotti, G. *et al.* Sediment eddy diffusivity in meandering turbulent jets: Implications for levee formation at river mouths. *Journal of Geophysical Research: Earth Surface* **118**, 1908–1920 (2013).

39. Canestrelli, A., Nardin, W., Edmonds, D., Fagherazzi, S. & Slingerland, R. Importance of frictional effects and jet instability on the morphodynamics of river mouth bars and levees. *Journal of Geophysical Research: Oceans* **119**, 509–522 (2014).

40. Yuill, B. T., Khadka, A. K., Pereira, J., Allison, M. A. & Meselhe, E. A. Morphodynamics of the erosional phase of crevasse-splay evolution and implications for river sediment diversion function. *Geomorphology* **259**, 12–29 (2016).

41. Rousseau, Y. Y., Van de Wiel, M. J. & Biron, P. M. Simulating bank erosion over an extended natural sinuous river reach using a universal slope stability algorithm coupled with a morphodynamic model. *Geomorphology* **295**, 690–704 (2017).

42. Norman, L. M. *et al.* Quantifying geomorphic change at ephemeral stream restoration sites using a coupled-model approach. *Geomorphology* **283**, 1–16 (2017).

43. French, J. Critical perspectives on the evaluation and optimization of complex numerical models of estuary hydrodynamics and sediment dynamics. *Earth Surface Processes and Landforms: The Journal of the British Geomorphological Research Group* **35**, 174–189 (2010).

44. French, J. Airborne lidar in support of geomorphological and hydraulic modelling. *Earth Surface Processes and Landforms: The Journal of the British Geomorphological Research Group* **28**, 321–335 (2003).

45. Son, M. & Hsu, T.-J. The effects of flocculation and bed erodibility on modeling cohesive sediment resuspension. *Journal of Geophysical Research: Oceans* **116** (2011).

46. Xie, D. *et al.* Morphodynamic modeling of a large inside sandbar and its dextral morphology in a convergent estuary: Qiantang estuary, china. *Journal of Geophysical Research: Earth Surface* **122**, 1553–1572 (2017).

47. Van Der Wegen, M., Dastgheib, A., Jaffe, B. E. & Roelvink, D. Bed composition generation for morphodynamic modeling: Case study of San Pablo Bay in California, USA. *Ocean Dynamics* **61**, 173–186 (2011).

48. Ralston, D. & Geyer, W. Sediment transport time scales and trapping efficiency in a tidal river. *Journal of Geophysical Research: Earth Surface* **122**, 2042–2063 (2017). URL

49. Ganju, N. K., Schoellhamer, D. H. & Jaffe, B. E. Hindcasting of decadal-timescale estuarine bathymetric change with a tidal-timescale model. *Journal of Geophysical Research: Earth Surface* **114** (2009).

50. Luan, H., Ding, P., Wang, Z. & Ge, J. Process-based morphodynamic modeling of the yangtze estuary at a decadal timescale: Controls on estuarine evolution and future trends. *Geomorphology* **290**, 347–364 (2017). URL

51. Davies, A. & Robins, P. Residual flow, bedforms and sediment transport in a tidal channel modelled with variable bed roughness. *Geomorphology* **295**, 855–872 (2017).

52. Hibma, A., Stive, M. & Wang, Z. Estuarine morphodynamics. *Coastal Engineering* **51**, 765–778 (2004). URL

53. Guo, L., van der Wegen, M., Roelvink, D. & He, Q. Exploration of the impact of seasonal river discharge variations on long-term estuarine morphodynamic behavior. *Coastal Engineering* **95**, 105–116 (2015). URL

54. Mariotti, G. & Canestrelli, A. Long-term morphodynamics of muddy backbarrier basins: Fill in or empty out? *Water Resources Research* **53**, 7029–7054 (2017). URL

55. Schwarz, C. *et al.* Impacts of salt marsh plants on tidal channel initiation and inheritance. *Journal of Geophysical Research: Earth Surface* **119**, 385–400 (2014). URL

56. Horstman, E., Dohmen-Janssen, C., Bouma, T. & Hulscher, S. Tidal-scale flow routing and sedimentation in mangrove forests: Combining field data and numerical modelling. *Geomorphology* **228**, 244–262 (2015). URL

57. Dissanayake, D. *et al.* Modelling morphodynamic response of a tidal basin to an anthropogenic effect: Ley bay, east frisian wadden sea - applying tidal forcing only and different sediment fractions. *Coastal Engineering* **67**, 14–28 (2012). URL

58. Hu, K., Chen, Q., Wang, H., Hartig, E. & Orton, P. Numerical modeling of salt marsh morphological change induced by hurricane sandy. *Coastal Engineering* **132**, 63–81 (2018). URL

59. Rossi, V. M. *et al.* Impact of tidal currents on delta-channel deepening , stratigraphic architecture , and sediment bypass beyond the shoreline. *Geology* **44**, 927–930 (2016).

60. Hajek, E. A. & Edmonds, D. Is river avulsion style controlled by floodplain morphodynamics? *Geology* **42**, 199–202 (2014).

61. Geleynse, N. *et al.* Controls on river delta formation: insights from numerical modelling. *Earth and Planetary Science Letters* **302**, 217–226 (2010).

62. Van de Lageweg, W. I. & Feldman, H. Process-based modelling of morphodynamics and bar architecture in confined basins with fluvial and tidal currents. *Marine Geology* **398**, 35–47 (2018). URL `https://doi.org/10.1016/j.margeo.2018.01.002`.

63. Hibma, A., De Vriend, H. & Stive, M. Numerical modelling of shoal pattern formation in well-mixed elongated estuaries. *Estuarine, Coastal and Shelf Science* **57**, 981–991 (2003).

64. Lotsari, E., Wainwright, D., Corner, G. D., Alho, P. & Käyhkö, J. Surveyed and modelled one-year morphodynamics in the braided lower Tana River. *Hydrological Processes* **2716**, 2685–2716 (2014).

65. Braat, L., Kessel, T. v., Leuven, J. R. & Kleinhans, M. G. Effects of mud supply on large-scale estuary morphology and development over centuries to millennia. *Earth Surface Dynamics* **5**, 617–652 (2017).

66. Van der Vegt, H., Storms, J. E. A., Walstra, D. J. R. & Howes, N. C. Can bed load transport drive varying depositional behaviour in river delta environments ? *Sedimentary Geology* **345**, 19–32 (2016). URL `http://dx.doi.org/10.1016/j.sedgeo.2016.08.009`.

67. Edmonds, D. A. & Slingerland, R. L. Significant effect of sediment cohesion on delta morphology. *Nature Geoscience* **3**, 105–109 (2009). URL `http://dx.doi.org/10.1038/ngeo730`.

68. Temmerman, S. *et al.* Vegetation causes channel erosion in a tidal landscape. *Geology* **35**, 631–634 (2007).

69. Ridderinkhof, W., de Swart, H. E., van der Vegt, M. & Hoekstra, P. Influence of the back-barrier basin length on the geometry of ebb-tidal deltas. *Ocean dynamics* **64**, 1333–1348 (2014).

70. Van Leeuwen, S., Van der Vegt, M. & De Swart, H. Morphodynamics of ebb-tidal deltas: a model approach. *Estuarine, Coastal and Shelf Science* **57**, 899–907 (2003).

71. Sanyal, J. Predicting possible effects of dams on downstream river bed changes of a himalayan river with morphodynamic modelling. *Quaternary International* **453**, 48–62 (2017).

72. Van Rijn, L., Walstra, D. & Ormondt, M. v. Description of transpor2004 and implementation in delft3d-online. *Z3748* (2004).

73. Engelund, F. & Hansen, E. A monograph on sediment transport in alluvial streams. *Technical University of Denmark Ostervoldgade 10, Copenhagen K.* (1967).

74. Meyer-Peter, E. & Müller, R. Formulas for bed-load transport. In *IAHSR 2nd meeting, Stockholm, appendix 2* (IAHR, 1948).
