# Supplementary material for: Critical dependence of morphodynamic models of fluvial and tidal systems on empirical downslope sediment transport
Source: Nat Commun. 2019 Oct 25;10:4903. doi: 10.1038/s41467-019-12753-x (PMC6961408; doi:10.1038/s41467-019-12753-x)
Supplement: Supplementary file 2 — Peer Review File [file 41467_2019_12753_MOESM2_ESM.pdf]

Reviewers' comments:

Reviewer #1 (Remarks to the Author):

I found this manuscript very interesting and the outcomes relevant and of interest to estuarine-fluvial morphodynamic community. The study shows how relevant the sediment transport parameterization and the transverse bed-slope parameter are as regards the prediction of morphodynamic evolution of fluvial and tidal systems. The morphodynamic evolution effects of the sediment transport parameterization has been suggested previously (but not analyzed in detail) and the effect of the transverse bed-slope parameter is novel.

The authors are right when stating that this term is used to diffuse the morphodynamic evolution and that there is not a clear way to select the calibration coefficients. This manuscript shows how relevant the selection of this coefficient is and how an incorrect choice of such values can create incorrect morphodynamic evolution predictions. It also shows how different combinations of slope transport formulas and sediment predictors produce very different morphological results.

My main “concern” with this manuscript is that the outcomes are applicable to sandy systems (medium-coarse sand) in which the bedload is the dominant sediment transport mechanisms. Although the formula of Van Rijn accounts for suspended load and bedload, many of the processes that are relevant for suspended sediment are not accounted for in this formula. How do these additional bed slope effects affect systems in which sediment suspension is the main sediment transport mode? Systems in which suspended sediment transport is dominant is not the focus of this study but it is not clearly indicated in the manuscript (although it is mentioned). For sandy environments, does the d50 or the selected morphodynamic factor affect the results? Does the “morphodynamic calibration” need to depend on the morphological acceleration factor and d50 that is being used or not?

I think the outcomes of this manuscript are relevant and should help morphodynamic modelers to make better choices about how to calibrate their models. Specific comments include:

1) I do not completely agree with one of the statements of the introduction section, which states that often roughness parameters of grain size distributions are tuned to converge to an stable morphology. The roughness parameter or the friction coefficient should be verified with hydrodynamic measurements and the initial grain size distribution is given by the initial setting. I would rephrase this sentence.

2) I do not completely understand the x axis in Figure 3: “Increasing scale of the processes studied”; there is not a consistent increase of the scale here… but maybe I missed something.

Reviewer #2 (Remarks to the Author):

The paper entitled “Critical dependence of morphological models of fluvial and tidal systems on empirical downslope sediment transport” addresses the way in which scientists currently apply computational models to study and forecast morphodynamics. In particular, it presents a problem in the current calibration technique of amplifying downslope sediment transport, investigates the problem’s cause, identifies a knowledge gap and presents a temporary solution while the knowledge gap remains unresolved.

Novelty, scientific and societal importance: The results are novel and make an important (and overdue) contribution to the rapidly growing field of morphodynamic modelling. The fast growth of this research field can be attributed to its application to studying and forecasting developments in densely populated areas such as river valleys, coastal plains and deltas, which are at risk from the effect of climate change and sea level rise.

Technically quality and strength of evidence for conclusions: The authors display a detailed understanding of the theory and conducted an elaborate study of exiting literature to frame the problem. As far as I can tell, the data is technically sound. However, the presentation of the results is not always methodical and/or clear. It is my opinion that, while this manuscript contains all the ingredients of a truly great paper, the presentation for the results should be improved and the link between the results and the conclusions strengthened. In the discussion and results sections, much clearer reference should be made to the relevant figures and results supporting the claims. This would ensure that conclusions do not overstate the results, making the paper stronger.

Recommendation: This work provides an important contribution to the geoscience community and should be published soon, as it can have a significant impact on how we parameterize morphodynamic models in the short term and points to a knowledge gap that needs to be addressed by the scientific community in the medium term. The authors are critical of techniques that have been effective at modelling of decade-scale morphological changes and sediment transport. It is therefore imperative that the conclusions are delivered in a clear and convincing fashion to maximize its impact. It is my opinion that this is not yet the case but that it can be addressed fairly simply. I have provided suggestions on how the manuscript could be improved by indicating where I found gaps in the reasoning or where the text or figures were unclear to me. It is my recommendation that with these improvements, this paper should be accepted for publication.

Kind Regards

Helena van der Vegt.

Reviewer #3 (Remarks to the Author):

The manuscript by Baar et al. deals with models simulating the morphodynamics of fluvial and tidal systems. The problem posed by the authors is extremely relevant: is the sediment transport approach used in essentially all numerical models fundamentally flawed? The authors have a case since model sensitivity to the downslope sediment transport is rarely reported or discussed in detail. Still, as the authors show, the present approach (exaggerating the gravity-driven component of sediment transport) can lead to artificial cross-sectional shapes and makes the model impossible to calibrate. The problem is real and the authors should be praised for tackling the issue and for showing so many examples supporting their arguments. I have little doubt the manuscript will be of interest to many. At the same time, the manuscript is very long and dense and then there is the appendix, which is even longer than the manuscript itself. I am not sure there is much that can be done and it is all interesting material.

My main issue is that the fundamental problem remains the prediction of sediment transport. Both the sediment transport predictor and the slope effect parametrization contain large uncertainties (I do appreciate the advance on the slop-term parameterization from Baar et al.). The results, as also shown by the authors, are a function of the choice of sediment transport predictor. It is difficult to generalize when sediment transport predictors are so different. This should be indicated. I appreciate that the problem is qualitatively similar for sediment transport predictors but quantitatively results are likely to be different for other predictors.

Also, there is work (Lanzoni and D'Alpaos, JGR 2015; Xu et al., JGR 2018) showing that a balance between the flow and the two sediment transport terms leads to the emergence of channels with a cross-sectional geometry in line with observations, and such configuration is an equilibrium one. While the microscopic details (eg, the sediment transport predictor) are certainly wrong, the macroscopic simulated behaviour of the system is consistent with observations and is an equilibrium one. Although simplified, I would say that the choices made in the above studies (there's more, primarily from University of Padua researchers) are far from "arbitrary" (page 25). The transport rates change enormously within a channel from the incipient stages to the equilibrium configuration (the authors could have shown this, also for a the simplified case of the formation of a single channel). This aspect is not discussed in the manuscript but it is certainly relevant.

The above points are not strictly criticisms but rather comments stimulated by the results presented. I think addressing the above points could enrich the discussion without affecting the relevance and impact of the work.

Overall, this is an excellent and stimulating piece of work and I would certainly like to see it published.

Response to reviewers:

Reviewer #1 (Remarks to the Author):

I found this manuscript very interesting and the outcomes relevant and of interest to estuarine-fluvial morphodynamic community. The study shows how relevant the sediment transport parameterization and the transverse bed-slope parameter are as regards the prediction of morphodynamic evolution of fluvial and tidal systems. The morphodynamic evolution effects of the sediment transport parameterization has been suggested previously (but not analyzed in detail) and the effect of the transverse bed-slope parameter is novel. The authors are right when stating that this term is used to diffuse the morphodynamic evolution and that there is not a clear way to select the calibration coefficients. This manuscript shows how relevant the selection of this coefficient is and how an incorrect choice of such values can create incorrect morphodynamic evolution predictions. It also shows how different combinations of slope transport formulas and sediment predictors produce very different morphological results.
We thank the reviewer for his/her comments.

My main “concern” with this manuscript is that the outcomes are applicable to sandy systems (medium-coarse sand) in which the bedload is the dominant sediment transport mechanisms. Although the formula of Van Rijn accounts for suspended load and bedload, many of the processes that are relevant for suspended sediment are not accounted for in this formula. How do these additional bed slope effects affect systems in which sediment suspension is the main sediment transport mode? Systems in which suspended sediment transport is dominant is not the focus of this study but it is not clearly indicated in the manuscript (although it is mentioned). For sandy environments, does the d50 or the selected morphodynamic factor affect the results? Does the “morphodynamic calibration” need to depend on the morphological acceleration factor and d50 that is being used or not?
The sediment mobility in our models is such that suspended load is important, and that is indeed calculated separately in van Rijn. It is not entirely clear which important suspension processes the reviewer refers to. We now mentioned lateral diffusion of suspended sand in the discussion of our manuscript, and we ignore, for the present, suspension of cohesive sediments because that indeed requires many more processes such as flocculation, hindered settling in near-bed fluff layers, cohesion and salinity effects. However, as far as we know, none of these processes are affected by bed slope. We now mention in the introduction and in the methods that we treat suspension of sand in the manuscript, while we do not treat mud.

The effect of sand grain size is twofold. First, the transport relations, including the suspended transport, depend directly on grain size, ultimately because fluid forces are balanced by total mass of the particles in the transport process. However, a second effect is here important. One of the main causes of the unrealistic incision in morphodynamic models is that the processes that are relevant for suspended sediment are not accounted for and therefore there are less slope effects acting on suspended sediment transport. Therefore, if there is more sediment transported in suspension, there is relatively less bed load transport that could counteract unrealistic grid-size dependent incision. In this case, the transverse slope parameter needs to be increased to deflect more sediment downslope. Therefore, assuming a certain limited range of velocities occurring in natural systems (Savenije) such that finer sediment leads to higher mobility (Parker) and a thus higher proportion of suspended transport, the slope effect that is needed in numerical models to obtain dynamic channels and realistic morphology indirectly depends on D50. We explain this in the discussion and we now mention more clearly that `slope’ processes on suspended transport are unknown and should be studied further how to implement this in the models. Additionally, we now mention in the discussion model studies of systems in which suspended sediment transport is dominant. It should be noted that these points do not qualitatively influence the recommendations at the end of the

paper of which predictor to use in combination with a specific slope parametrization, pending fundamental sediment transport research and model improvements.

An analyses of the effect of the morphological acceleration factor is not included in this manuscript, since our test runs indicated that this did not have an effect on the general braided river model characteristics analysed here. However, it is expected that it will have an influence on the morphology in tidal systems and also possibly when there are multiple grain sizes present. However, there are other processes that also might have an influence on the magnitude of the slope parameter that is needed to counteract extreme incision, such as the choice of roughness predictor and diffusivity. None of these parameters have such a large effect that they solve the underlying problem of grid size-dependent incision, and, inversely, the acceleration factor did not have such a large effect as to exacerbate the incision problem.

I think the outcomes of this manuscript are relevant and should help morphodynamic modelers to make better choices about how to calibrate their models. Specific comments include:
1) I do not completely agree with one of the statements of the introduction section, which states that often roughness parameters of grain size distributions are tuned to converge to an stable morphology. The roughness parameter or the friction coefficient should be verified with hydrodynamic measurements and the initial grain size distribution is given by the initial setting. I would rephrase this sentence.
We rephrased this sentence in the introduction. We mean that any morphodynamic model with default parameters will show unstable morphology, and in practice modellers choose a sediment transport predictor, roughness predictor, slope effect etc. that will lead to a morphology that fits with their model objectives. Specifically, unrealistic incision is often counteracted by increasing the slope effect and/or adding coarser sediment fractions, and this is influenced by the choice in roughness predictor. For example, using a constant Chezy roughness will lead to channels that are less deep compared to channels in models with the Ks roughness height that results in varying roughness with water depth (Kleinhans et al. 2008 in WRR). We agree with the reviewer that, ideally, the magnitude of the friction coefficient is verified with hydrodynamic measurements and the grain size matches the system that is modelled. However, in practice this is often not the case whether the reviewer and authors agree with that is a moot point. Moreover, the problem is that even for the most optimistic choices of roughness and grain size mixtures (Baar et al. 2019 Sedimentology), the models will still show unrealistic incision, and in many calibrated models like the Western Scheldt model (Supplement 5) the transverse bed slope parameter still needs to be increased up to two orders of magnitude larger than the default value to get a stable morphology.

2) I do not completely understand the x axis in Figure 3: “Increasing scale of the processes studied”; there is not a consistent increase of the scale here… but maybe I missed something.
We now see that ‘scale’ was not the right word, and we changed the axis title to: “Increasing complexity of the processes studied”. We start with looking at the balance between incision and transverse sediment transport (left side) at a simple channel cross-section. Then, we look at a larger scale and look at the influence of increasing slope effects on braiding index and bar and channel dimensions (middle part of the figure), which have a more complicated feedback with flow and sediment transport. Lastly, we look at slope effects in the calibrated model of the Western Scheldt estuary that is more complex than a simple balance (right side), because the morphology and observed change in slopes depend on tides and develop over a much larger range of conditions of depth, sediment mobility and sediment transport nonlinearity than the other systems.

Reviewer #2 (Remarks to the Author):

The paper entitled "Critical dependence of morphological models of fluvial and tidal systems on empirical downslope sediment transport" addresses the way in which scientists currently apply computational models to study and forecast morphodynamics. In particular, it presents a problem in the current calibration technique of amplifying downslope sediment transport, investigates the problem's cause, identifies a knowledge gap and presents a temporary solution while the knowledge gap remains unresolved.

Novelty, scientific and societal importance: The results are novel and make an important (and overdue) contribution to the rapidly growing field of morphodynamic modelling. The fast growth of this research field can be attributed to its application to studying and forecasting developments in densely populated areas such as river valleys, coastal plains and deltas, which are at risk from the effect of climate change and sea level rise.

Technically quality and strength of evidence for conclusions: The authors display a detailed understanding of the theory and conducted an elaborate study of exiting literature to frame the problem. As far as I can tell, the data is technically sound. However, the presentation of the results is not always methodical and/or clear. It is my opinion that, while this manuscript contains all the ingredients of a truly great paper, the presentation for the results should be improved and the link between the results and the conclusions strengthened. In the discussion and results sections, much clearer reference should be made to the relevant figures and results supporting the claims. This would ensure that conclusions do not overstate the results, making the paper stronger.

Recommendation: This work provides an important contribution to the geoscience community and should be published soon, as it can have a significant impact on how we parameterize morphodynamic models in the short term and points to a knowledge gap that needs to be addressed by the scientific community in the medium term. The authors are critical of techniques that have been effective at modelling of decade-scale morphological changes and sediment transport. It is therefore imperative that the conclusions are delivered in a clear and convincing fashion to maximize its impact. It is my opinion that this is not yet the case but that it can be addressed fairly simply. I have provided suggestions on how the manuscript could be improved by indicating where I found gaps in the reasoning or where the text or figures were unclear to me. It is my recommendation that with these improvements, this paper should be accepted for publication.

Kind Regards

Helena van der Vegt.

We thank the reviewer for a thorough review of the text and figures. We improved our definitions of environment, perturbations and total sediment transport versus transverse sediment transport, and we added more references to figures and relevant supplements throughout the manuscript. We implemented the other suggestions for clarification of the text and figures where relevant and below we comment on the points (also those made in the manuscript PDF by the reviewer) that need more explanation. For clarification, we grouped some of the comments that are related to the same subject.

We grouped the following comments, because they all relate to the relative magnitude of the transverse sediment transport compared to streamwise sediment transport, and the effect of suspended sediment transport on this relative magnitude, which determines the balance between incision and reduction thereof by transverse sediment transport.

General comment:

- There is no comparison of the transverse sediment fluxes with overall sediment transports nor the partitioning of transported sediment transported into suspended and bedload. These values will also vary significantly between then models with different slope parameters and there is no way to tell whether these or slope

Comments from result section 1 "Effects on general morphology of combinations of sediment transport predictor and slope parameterization":

- "The most worrying conclusion is that the braided river model with the EH transport predictor and an αK of 0.7 (slope effect = 7) has similar morphology as with an αI of 1 (slope effect = 1) (Fig. 4), but has seven times larger transverse sediment fluxes, since the slope effect is seven times larger."
  It the transverse transport is much smaller than the total transport, then a factor 7 different between the two would not really be that worrying at all. It would be insightful to put this into perspective by also providing the overall transport for comparison or analysing a ratio of the transverse vs. overall transport in each cell. This would make the result a lot stronger.
- "When using another sediment transport predictor this difference is even larger, since a hundred times larger slope effect is needed in the model with the VR transport predictor to get similar bed slopes as in the model with the EH transport predictor. In this example it was not possible to get a realistic morphology in combination with the KF slope parameterization." In the VR transport formula sediment is transported as suspended load and bedload. Again, to quantify the importance of this calibration, the increase in transverse sediment flux should be compared to the total sediment flux (transverse and along stream) to quantify its importance on morphological development. Quite possibly, the VR sediment transport predictor has a very small proportion of sediment assigned to be transported as bedload in these scenarios.

Comments from result section 2 "Imbalance between incision and transverse sediment transport at the channel scale":

- "The unrealistic channel erosion in numerical models suggest an imbalance between channel incision and transverse sediment transport. Therefore, the over-deepening of channels can be the result of either of these two processes."
  What about flow parallel bedload transport in general in this balance? Surely this can also balance channel incision?
- "For the sediment transport predictor of Van Rijn, we only take the bed load part into account in the analytical model, since in Delft3D slope effects only act on the bed load". The second part of the sentence does not support the first part, since the suspended load also deposits eventually - when concentration becomes larger than the equilibrium concentration) thereby counteracting incision. If the suspended load is much larger than the bed load, then surely the SL cannot be ignored?

Comments from the discussion:

- "Practically, this means that it is impossible to have both realistic sediment transport rates and morphology in the same model study," This is an overstatement of the results. The paper currently discusses only transverse sediment transport rates and does not put this into context with overall sediment transport rates and therefore cannot make this claim based on the presented data.

- "for certain do not produce realistic sediment transport rates and direction," again, does the importance of this not depend on the partitioning bedload vs. suspended load and the partitioning downslope vs overall transport in the simulation?
- 'it is better to calibrate the model on for example migration rates of channels instead of bed levels." This depends of the timescale modelled, the rate of change in the morphology within that timescale and the size if transverse vs. overall sediment transport within that timescale.

The most important message is that the increase of slope parameter changes the relative amount of transverse sediment transport compared to streamwise sediment transport linearly, regardless the size of the streamwise vector. In case of an increase in slope effect from 1 to 7, this means 7 times as much sediment transport in the transverse direction. Therefore, the direction of the total transport vector will change on slopes with the same magnitude. This is also illustrated with the schematic drawing of the transport vectors in figure 2. We now explained this better in the first section of the results that describes the general change in morphology in the braided river model. Slope parameters outside the calibration range therefore "for certain do not produce realistic sediment transport rates and direction" compared to the transport vector that would be present in that same grid cell with the default slope parameter.

Figure 7 in section 4 of the results quantifies the effect of this relative change in transverse sediment transport and change in direction on bar and channel properties, which shows a large difference between models with increasing slope effect. Comparison of transverse fluxes with streamwise fluxes for each cell will not make the results clearer however, because the transverse sediment flux depends on slope and sediment mobility and therefore varies throughout the model and will adapt to the resulting change in morphology. This will therefore be difficult to interpret correctly for the reader. The cumulative slope distributions (fig. 4 and S.11) show that there is a large range in slopes throughout the models and therefore there are cells in the model that have a large relative transverse sediment flux compared to the streamwise vector and cells with a lower ratio.

We explain in the discussion that the magnitude of the slope factor that is needed to get realistic channel dynamics and morphology depends on the amount of bed load that is available to be deflected. A small amount of bedload transport can indeed be the result of large amount of suspended sediment, as we also explain in the discussion. However, this means that if you have a small bed load transport rate and an alpha_bn of e.g. 30, the direction of the bedload vector and the amount of transverse sediment transport compared to streamwise sediment transport is not realistic.

We agree that the impact it has on conclusions made from a model depend on the timescale that is modelled and the amount of sediment transport, which determines how fast the results of unrealistic transport vectors or unrealistic morphology become problematic. We now mention this more clearly in the last section of the discussion with the example of the Western Scheldt. The Western Scheldt model shows the difference in transport vector magnitude and direction on the same slopes even for a short time period (Fig S.11). Therefore, regardless of the time scales, the change of direction of the transport vector by increasing the slope effect is a problem that people should be aware of. To be precise, when the morphology in a model is realistic, the slope effects are unrealistically increased, which leads to transport vectors that are unrealistic in magnitude and direction. Therefore, the statement "this means that it is impossible to have both realistic sediment transport rates and morphology in the same model study" is unfortunately not an overstatement but the main reason why this study was, in the words of one reviewer and a PhD committee member of the first author, long overdue.

We grouped the following comments (<u>all from the discussion</u>), because they all relate to the question whether the severe incision depends on a positive feedback that can be prevented by starting with a morphology that is closer to equilibrium:

- "With increasing slope effect, this grid size-dependent incision decreases and with a sufficiently large slope effect there is no trend with grid size" We need to know what happens over time – do these incisions reach an equilibrium depth or do they continue to grow indefinitely? Does feedback of incision have anything to do with how far the initial morphology is from the equilibrium situation? Does the model get trapped in a feedback loop because the changes required to reach an equilibrium are so large that velocities become too high as the model strives to reach its equilibrium situation? Is the increase in transverse sediment transport so successful as calibration parameter because it buffers large changes in morphology for long enough so that the model can reach a dynamic equilibrium state?
- "The cause of this imbalance is twofold. Firstly, the amount of channel incision is highly depended on grid size, suggesting strong numerical effects" How does the difference between an equilibrium situation and the initial situation effect this? Could it be that a lack of morphological "spin-up" causes this feedback?
- "depends on if a model needs to be laterally dynamic or not," if a model starts far from its equilibrium morphology, it may initially cause a deep incision which limits its natural lateral variability to such an extent that it is not possible to later overcome this.
- "the effect of slope parametrization might seem less obvious due to often smaller or constrained model domains and shorter run time." Or perhaps less important when the bedload constitutes a small proportion of the overall transport and the starting morphology is close to the dynamic equilibrium morphology of the system?

The questions raised here boil down to the question whether the morphological models can produce more than one equilibrium morphology, namely one that is incision gone out of hand, and one that did not go out of hand because the initial model condition was sufficiently close to one of the two equilibrium conditions. However, we never found indications that the model equilibrium depended in this sense on the initial conditions (except where this means a static equilibrium that is only reached because the sediment is no longer mobile). This is in fact good news for morphology and for modelling, and is generally supported by physical scale experiments in the past. It also means that morphological spin-up (cold-starting) from a flat bed rather than close-to-equilibrium morphology does not affect the final equilibrium.

This is illustrated by the Western Scheldt model, which started with measured bathymetry to mimick the practice of modelling and for reasons of computational cost, and the results in fig S.8 and S.11 are after ten years, which is the time period that the model is used for in other studies. After ten years, we see that default values of the slope effect lead to slopes that are too steep even though the model started form the measured morphology. When the model is run for longer, the slopes start to steepen further and experience the positive feedback that leads to unrealistic incision, and this is also the case for the models with increased slope effect that were closer to the input morphology after ten years. Since these models that are based on existing morphologies start closer to equilibrium and usually run for shorter time periods, the imbalance between incision and downslope sediment transport is less obvious, but this is not the solution for the problem. On the other hand, with a sufficiently unrealistic slope effect and starting with a plane bed or a measured bathymetry, the same reasonable morphologies were obtained after centuries by van der Wegen and Roelvink (2012, Geomorphology). We now added this explanation to the last section of the discussion where we describe models that are designed to represent existing morphology.

Below, we answer individual comments:

- Introduction paragraph 2: 'up to a 100 times higher than physically correct".
  While strictly true, this overstates the problem slightly. There are only a few studies in Supplement 6 which use 10-100 value for alphabn. The majority of the studies use 1-2, within the calibration range. Which of these studies are calibrated to read world morphological developments compared to synthetic models not calibrated to measurements in natural systems. Are calibrated models more likely to have adjusted alphabn values?

The literature inventory (Supplement 6) shows that the magnitude of the slope effect depends on the environment that is modelled, and all of the models with an environments that depend on a balance between erosion and deposition, and therefore needed a dynamic system, needed a slope parameter outside the calibration range (i.e. larger than 2). We suspect that many models that did not mention the slope parameter needed a larger slope parameter than the calibration range to get a realistic morphology and implicitly admit this by reference to earlier work. Therefore we believe that we are not overstating the problem by saying that slope parameters are adjusted *up to* a 100 times.

Indeed, models that have more realistic bar and channel patterns have increased slope parameters outside the calibration range, so that includes calibrated models but also idealized models that had the objective to look at a dynamic systems with e.g. natural bar dimensions and braiding index.

- First statement in the results section: "All five models (Fig. 3) generally showed deep incision and over steepened morphology with physically correct slope effects, leading to deep and narrow channels, a higher number of channels and shorter bars than typically observed in nature".
  I cannot find results supporting the above statement are not provided for all 5 models (also not in the supplement 3) whereby the above statement may be an overstatement of the results. Could the authors refer to where the data on which this statement is based is presented for the 5 models (which figures or tables). Additionally, how is "over steepened morphology" determined for the 5 models? Especially for the which are not representing a specific natural system? Perhaps this can be resolved by listing the compatible natural system (now only in methodology) for each of the 5 models in figure 3. e.g. Schematised Old Rhine mouth at Leiden etc.

We now refer to the relevant figures in the supplement and changed the word 'oversteepened' to 'steep'. Oversteepened indeed suggests we compared it to measured morphology which is only the case in with the Western Scheldt model. It is however visible in the cumulative distributions of slopes of all models that the morphology is much steeper with default values (fig. 4 for the braided river model and fig. S.8 in the supplement for the other models). Furthermore, we show with the DEMs of all models (fig 4 and fig S.6 and S.7 in the supplement) and further analyses of braiding index, channel depth and bar lengths (fig 7) that all the models did not show natural morphology with default slope effects.

- Figures 4: Does this figure show transverse slopes based on the flow direction for each cell in the domain? For all time intervals or only for the "final" morphology? How does this distribution change over time? Is this distribution of slopes an equilibrium/characteristic distribution of slopes for this model or simply a snapshot in time? That could significantly impact the relevance of this result.

Figure 4 shows the slopes for each cell in the domain only for the final morphology, which we now clarify in the caption. All runs started at a flat bed and during the development of the morphology the slope distributions begin to deviate until the result plotted in the graph in figure 4.

- Results section 2: "The unrealistic channel erosion in numerical models suggest an imbalance between channel incision and transverse sediment transport. Therefore, the over-deepening of channels can be the result of either of these two processes."What about flow parallel bedload transport in general in this balance? Surely this can also balance channel incision?

Yes channel erosion or deposition is also determined by the change in streamwise sediment transport, but, no, this is not sufficient to balance the erosion tendency, which requires increasing the slope effect.

- Results section 2: *"The most important negative feedback on channel formation is sediment transport deflection on the side slopes towards the center of the channel under the influence of gravity"*
  This is true in single grain size fractions in scenarios with a mixture of grain sizes? Wat about when an armouring layer builds up at the channel base as the sediment mixture undergoes sorting over time?

Fundamentally this is still a balance between incision and downslope sediment transport when the channel starts forming, only sediment is sorted along the slope that develops. However this can lead to armouring that prevents erosion. Some studies therefore suggest that the severe incision in the model is caused by the use of uniform sediment instead of a sediment mixture, which is at least partly the tail wagging the dog given active layer model limitations (see for review Baar et al. 2019, Sedimentology). However, for realistic grain size mixtures active sediment sorting will not lead to different transverse slopes (Baar et al 2019, sedimentology). Our results show that the extreme gridsize-dependent incision with uniform sediment is not natural, and therefore, adding coarser sediment fractions to the model does not solve the problem of severe incision, but can mask it by resulting in a coarse, non-erodible bed layer that prevents erosion. We added this explanation to the first paragrapgh of the discussion.

- Results section 2: "Channels with lower ratios should theoretically show decaying perturbations, while models with higher ratios should have growing perturbations."
  Do you mean channels with lower equilibrium ratios, or channels with a lower ration that its calculated equilibrium? Even if that is clear I would still not be sure what this statement means because the definition of "growing" and "perturbation" is not clear in the text. Can perturbation be erosive or depositional or only erosive? Once that is resolved, could you also explain what supports this statement?

This terminology refers to mathematical stability analyses where the fastest-growing (periodic) perturbations predict bar and channel patterns. We now explain that this means roughly that wider and shallower channels develop bars while deeper and narrower do not, which is qualitatively supported by numerical modelling (Schuurman et al. 2012 JGR). This statement is supported by literature (Struiksma et al 1985, cited in the text). We now explained the meaning of perturbation better in the text.

- Results section 2: "For the sediment transport predictor of Van Rijn, we only take the bed load part into account in the analytical model, since in Delft3D slope effects only act on the bed load".
  The second part of the sentence does not support the first part, since the suspended load also

deposits eventually - when concentration becomes larger than the equilibrium concentration) thereby counteracting incision. If the suspended load is much larger than the bed load, then surely the SL cannot be ignored?

This specific sentence refers to the analytical model, which only calculates the balance over the cross-section, as we now clarify in the first paragraph of Supplement 2. In both models we assume that the sediment transport in streamwise direction is in equilibrium with the constant flow conditions. In the case of the numerical model, the transition from a dampening to growth of the perturbation was well-predicted, showing that the deposition of suspended sediment is clearly not enough to balance the absent slope effects on suspended sediment. Hence models with dominant suspended load need the high slope factor as also shown by our literature inventory. Papers that describe realistic morphology for systems with very high suspended sediment rates either had a large numerical diffusion to compensate for absent slope effects (as in Finel2D of Dam et al 2016) or needed an alpha_bn of 1000 (van Maren 2007 Sedimentology), which we now mention in the discussion.

- Discussion: No results from the western Scheldt model are presented in the results section, only in the supplement. Yet some very bold statements it made about its applicability in predicting dredging and dumping activity in the discussion section. If this is to be an important discussion point or point of criticism, then the supporting results also needs to be included in the results section rather than a supplementary.

We decided to put the results from the Western Scheldt model in the supplement, because we did not want the main text to be too long and complicated to follow. If the editor agrees we can put it in the main text. The conclusions made about calibrated models that are used for decision making also follow from the results plotted in the main text as follows. Different slope parameterizations lead to different magnitude and direction of sediment transport on the same slope (fig 2). Therefore, different slope parameterizations lead to different morphologies (fig. 4). It follows that a model calibrated on measured bathymetry will show different transport directions and magnitudes for different slope parameterizations (and transport predictors), which was also recognized by Reviewer 1 as a general outcome of this study.

Reviewer #3 (Remarks to the Author):

The manuscript by Baar et al. deals with models simulating the morphodynamics of fluvial and tidal systems. The problem posed by the authors is extremely relevant: is the sediment transport approach used in essentially all numerical models fundamentally flawed? The authors have a case since model sensitivity to the downslope sediment transport is rarely reported or discussed in detail. Still, as the authors show, the present approach (exaggerating the gravity-driven component of sediment transport) can lead to artificial cross-sectional shapes and makes the model impossible to calibrate. The problem is real and the authors should be praised for tackling the issue and for showing so many examples supporting their arguments. I have little doubt the manuscript will be of interest to many. At the same time, the manuscript is very long and dense and then there is the appendix, which is even longer than the manuscript itself. I am not sure there is much that can be done and it is all interesting material.

We thank the reviewer for his/her comments. We know it is a long paper, but as all reviewers acknowledge it is the entire set of very different environments that makes this work of general interest to a very wide community. We have done our best to improve readability on the basis of review comments and by putting some material in the supplements.

My main issue is that the fundamental problem remains the prediction of sediment transport. Both the sediment transport predictor and the slope effect parametrization contain large uncertainties (I do appreciate the advance on the slop-term parameterization from Baar et al.). The results, as also shown by the authors, are a function of the choice of sediment transport predictor. It is difficult to generalize when sediment transport predictors are so different. This should be indicated. I appreciate that the problem is qualitatively similar for sediment transport predictors but quantitatively results are likely to be different for other predictors.
We agree and generalize this point from our study of two very different predictors (Engelund-Hansen and Van Rijn). We now indicate more clearly in the discussion that the behaviour of sediment transport predictor in combination with slope parameterization is qualitatively similar in case of total load predictor or predictor with suspended load (advice in figure 8), but quantitatively the predicted sediment transport rate and dimensions of morphodynamic features will depend on the non-linearity of the transport predictor and other predictor-specific parameters.

Also, there is work (Lanzoni and D'Alpaos, JGR 2015; Xu et al., JGR 2018) showing that a balance between the flow and the two sediment transport terms leads to the emergence of channels with a cross-sectional geometry in line with observations, and such configuration is an equilibrium one. While the microscopic details (eg, the sediment transport predictor) are certainly wrong, the macroscopic simulated behaviour of the system is consistent with observations and is an equilibrium one. Although simplified, I would say that the choices made in the above studies (there's more, primarily from University of Padua researchers) are far from "arbitrary" (page 25). The transport rates change enormously within a channel from the incipient stages to the equilibrium configuration (the authors could have shown this, also for a the simplified case of the formation of a single channel). This aspect is not discussed in the manuscript but it is certainly relevant.
We agree that the choice in slope effect is not always arbitrary for the small amount of studies that mentions the effect of increasing the bed slope parameter, but for a large amount of the studies that does not mention the slope parameter it is.

The studies of Lanzoni & D'Alpaos and Xu et al. are dominantly erosional models with only suspended mud transport (see response to Reviewer 1). (We wonder to what degree their equilibrium morphology of a single, dead-ended channel is entirely constrained by the well-defined boundary conditions, the fixed model domain and the threshold for erosion). We now discuss model studies of systems in which suspended sediment transport is dominant. These studies show that mechanisms such as diffusion and dampening of turbulence are important for suspended sediment and should be studied further to implement realistically in morphodynamic models, since the absence of realistic slope effects on suspended sediment transport is one of the causes of the extreme channel incision.

The 'enormous' change of transport magnitude from incipient channel cutting on a shallow, flat surface, and equilibrium configuration is in general agreement with our findings of run-away erosion, but not relevant because the same morphology results when a near-equilibrium morphology is taken as initial condition (see response to Reviewer 2). We now explain this better at the first paragraph of the discussion by explaining the positive feedback that results in the unrealistically deep equilibrium channel, and we now comment on the effect of initial conditions on equilibrium morphology at the end of the discussion when we discuss models that are designed to represent existing morphology.

The above points are not strictly criticisms but rather comments stimulated by the results presented. I think addressing the above points could enrich the discussion without affecting the relevance and impact of the work.

Overall, this is an excellent and stimulating piece of work and I would certainly like to see it published.
Thank you.

REVIEWERS' COMMENTS:

Reviewer #1 (Remarks to the Author):

I found this manuscript very interesting and the outcomes novel, relevant and of interest to estuarine-fluvial morphodynamic community. The study shows how relevant the sediment transport parameterization and the transverse bed-slope parameter are as regards the prediction of morphodynamic evolution of fluvial and tidal systems. The morphodynamic evolution effects of the sediment transport parameterization has been suggested previously (but not analyzed in detail) and the effect of the transverse bed-slope parameter is novel.

The authors have addressed the main concern I had with the manuscript and have considered all my suggestions. The current version of the manuscript is very well written and well structured. I think this is a very good study and that it will have a great impact in the morphodynamic community. For these reasons I recommend the publication of the manuscript after the following minor editorial typos have been corrected:

Page 20, line 1: there is a "to" missing before started.

Page 26, line 10: there is missing a space before "and are not a solution,"

Page 26, line 18: there is missing a space between transportvectors.

Page 27, line 14: there is something wrong with the figure numbers and parenthesis.

Other than those minor typos, I think this is a great scientific contribution. I learned and enjoyed reading this manuscript.

Reviewer #2 (Remarks to the Author):

As mentioned in my initial review, the paper entitled “Critical dependence of morphological models of fluvial and tidal systems on empirical downslope sediment transport” provides an important contribution to the geoscience community by

- presenting a problem in the current calibration technique amplifying downslope sediment transport,

- investigates the cause of this problem,
- identifying a knowledge gap,
- and presenting a temporary solution while the knowledge gap remains unresolved.

The results are novel and make an important (and overdue) contribution to the rapidly growing field of morphodynamic modelling. The authors display a detailed understanding of the theory and conducted and thorough study of exiting literature to frame the problem. The data and methodology are technically sound.

I feel that the comments from the first review have been sufficiently addressed and it is my recommendation that this paper should be accepted for publication.

Kind Regards

Helena van der Vegt.

Reviewer #3 (Remarks to the Author):

I am satisfied with the reply from the authors to my comments. I also think this version of the manuscript has definitely improved.

I maintain this manuscript is relevant because, joining the discussion raised by reviewer 1, even though we should perform model calibration, there is a vast proliferation of studies that do not calibrate the models but still discuss temporal and spatial changes. The manuscript is relevant because it provides a clear assessment of the implications of "abusing" sediment transport formulations.
